# Acute clinical deterioration and consumer escalation: The understanding and perceptions of hospital staff

**DOI:** 10.1371/journal.pone.0269921

**Published:** 2022-06-16

**Authors:** Lisa Thiele, Arthas Flabouris, Campbell Thompson

**Affiliations:** 1 Faculty of Health and Medical Sciences, Adelaide Medical School, The University of Adelaide, Adelaide, South Australia, Australia; 2 Intensive Care Unit, Royal Adelaide Hospital, Adelaide, South Australia, Australia; 3 General Medicine Service, Royal Adelaide Hospital, Adelaide, South Australia, Australia; Massachusetts General Hospital, UNITED STATES

## Abstract

**Introduction:**

Consumer escalation systems allow patients and families to escalate concerns about acute clinical deterioration. Hospital staff can impact upon the success of this process. As part of evaluation processes within a Local Health Network, where a consumer escalation system was introduced in accordance with National requirements, we sought to explore clinicians’ understanding and perceptions of consumer escalation.

**Methods:**

Voluntary and anonymous staff surveys pre, and post, system introduction. Quantitative data was analysed using descriptive statistics, chi-square independence, and non-parametric independent samples median tests. Qualitative data was evaluated using content analysis and cross-referenced with quantitative responses.

**Results:**

Respondent’s (pre: 215; post: 89) area of work varied significantly between survey periods. Most agreed that patients/families have a sound knowledge of a patient’s typical health status (pre: 192/215 (89.3%); post 82/88 (93.2%)) and that patients/families should be encouraged to escalate concerns of deterioration to ward staff (pre: 209/212 (98.6%); post: 85/89 (95.5%)). Respondent perceptions of patient/family ability to recognise clinical deterioration varied. Staff agreement towards local response expectations decreased as the degree of clinical requirement increased. Staff concerns of increased workloads (pre: 90/214 (42.1%); post 12/72 (16.7%), p<0.001) and conflict generation (pre: 71/213 (33.3%); post: 7/71 (9.9%), p = 0.001) decreased significantly following system introduction. However, clinician perceptions of positive system effects also decreased (patient-staff rapport pre: 163/213 (76.5%); post: 38/72 (52.8%), p = 0.001; patient centred care pre: 188/214 (87.9%); post: 53/72 (73.6%), p = 0.012; patient safety pre: 173/214 (80.8%); post: 49/72 (68.1%), p = 0.077). Only 53% of respondents (pre: 112/213 (52.6%); post: 48/88 (54.5%)) perceived that patient/family have sufficient confidence to escalate concerns.

**Conclusion:**

Consumer escalation systems require staff support. Staff perceptions may indicate, and act as, barriers to the operation of consumer escalation processes. Further exploration in identifying and managing staff barriers is crucial to the success of consumer escalation.

## Introduction

Acute clinical deterioration may encompass a worsening in a patient’s physiological, cognitive, or mental state [1]. It is a dynamic situation [2] where early identification and response are essential to patient outcomes and safety [1, 3]. As health services are faced with mounting demands, an aging population, and a higher prevalence of comorbidities and illness acuity, the potential for hospitalised patients to experience clinical deterioration is continuing to increase [4]. The importance of enhancing knowledge in association with this topic can, therefore, not be underestimated.

Indications of clinical deterioration can be present for hours prior to serious adverse events in hospitalised patients [5, 6]. However, despite the introduction of clinician activated rapid response systems (RRS) [7] and track and trigger processes [8], delays, or failures, in staff response have still been identified in over 20% of cases of acute deterioration [9–12]. In reaction to such deficiencies and investigations of sentinel events, consumer escalation systems have emerged with the intention of increasing patient safety [13–15]. Consumer escalation systems are processes that allow hospital inpatients, their family, or carers a way to escalate their concerns about acute clinical deterioration [16].

Published literature relating to consumer escalation has emerged from the United States of America [17–22], Australia [23–27], the United Kingdom [28–30], New Zealand [31], and Singapore [32]. Initial work focused primarily upon describing system implementation within individual facilities, particularly in paediatric settings. However, limitations have been noted in the overall quality of studies [33]. Although further research is emerging with a greater focus on rigor and breadth [34], substantial gaps remain within the evidence base. Numerous models of consumer escalation exist and there is no consensus as to which is most effective [35] from either a patient or resource perspective. The ability of consumer escalation processes to achieve their underlying goals, particularly in relation to patient outcomes, has not been adequately determined [14, 35]. Questions and concerns also remain about potential barriers to effective system implementation [24], including barriers that may prevent consumer escalation systems from achieving their patient outcome related goals.

One potential barrier relates to the perceptions and actions of hospital staff, including those providing direct patient care through to management levels. Wider research has demonstrated that staff associated factors can critically impact upon the introduction, sustainability, and success of patient-focused interventions [36]. Yet, there has only been one prior study dedicated to examining clinician views (more precisely, medical staff views) of consumer escalation [21]. This study was also limited to a single paediatric hospital [21]. A further two studies included clinicians, as well as patients and family members, in interviews and focus groups to examine general barriers and facilitators to the implementation of consumer escalation processes, again, within a paediatric setting [24, 25]. The remaining papers that have broached this topic have either been small [17], a feasibility study [28], used retrospective data [23], provided only brief commentary [20, 22], or related to a study protocol [34]. Collectively, they highlight the need for further research of the potential impact of hospital staff upon consumer escalation processes, particularly amongst adult inpatients.

In recognition of this, we report on the outcomes of staff surveys completed as part of evaluation processes within an Australian Local Health Network (LHN) where a consumer escalation system was to be introduced in accordance with mandatory National requirements.

### Aims

The aims of the study were to explore medical, nursing, and allied health staff understanding and perceptions of consumer escalation for acute clinical deterioration in the hospital setting and to evaluate if staff knowledge and views changed after the introduction of a local consumer escalation system. It was anticipated that this knowledge would assist in identifying barriers that may impact upon the implementation and effectiveness of consumer escalation processes.

Based on literature review findings [35], three research questions were established to assist in achieving these study aims. The first question was to what extent do staff support the ability of the patient and their family to identify clinical deterioration and escalate concerns? The second question was what are the perceptions of staff towards responding to consumer concerns of deterioration? The final question was how do staff perceive consumer escalation systems and the impact of system introduction?

## Materials and methods

### Design

Cross sectional, anonymous, and voluntary staff surveys were completed as part of local evaluation processes. An associated EQUATOR CHERRIES checklist is provided within the supporting information (see S1 File).

### Setting and background

Surveys were collected across an Australian LHN incorporating five adult inpatient treatment sites (totalling greater than 1300 beds). Provided services included acute care, rehabilitation, mental health, and non-acute support for older individuals. Within this Network, an 800-bed quaternary adult acute public teaching hospital forms the primary site. The hospital has an established RRS and utilises a human-factors designed Rapid Detection and Response (RDR) observation chart. This chart is intended to support the detection of, and tiered response to, physiological deterioration [37]. The RRS provides two levels of response, namely a Medical Emergency Team (MET) and a Code Blue. A MET is initiated in response to predefined indicators of clinical deterioration as outlined within the RDR chart or in response to clinician concern of deterioration in a patient’s condition, even in the absence of a measurable trigger. A Code Blue is initiated in response to cardiac or respiratory arrest, a threatened airway, significant bleeding, or any concern of deterioration in a patient’s condition outside of the treating ward. In both a MET and Code Blue, the attending rapid response team (RRT) is medically staffed and led, and includes at least one critical care nurse. The responding team in a Code Blue also includes the additional support of a senior intensive care medical officer and a second critical care nurse.

**Fig 1:**
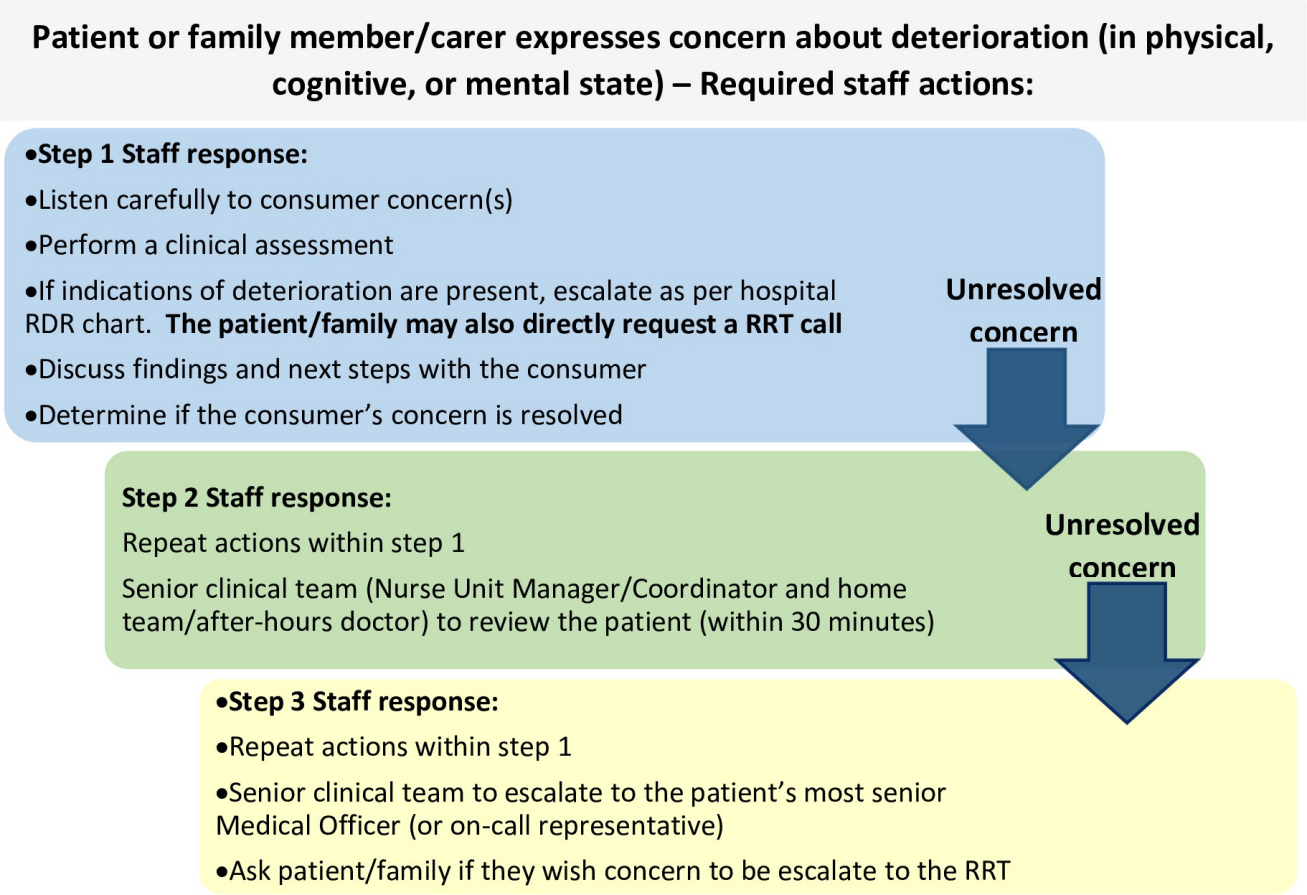
Local consumer escalation pathway for patient/family/carer concern of acute clinical deterioration.

Within Australia, it is a requirement of the National Safety and Quality Health Service Standards that all acute healthcare services have a system that permits patients, families, and carers to obtain assistance and escalate care if they are concerned about acute clinical deterioration in their own/family member’s condition [16]. A patient and family/carer escalation system was therefore introduced across the LHN in September 2019. At the time of collecting data, the system represented a step-wise, indirect consumer escalation model in which patients and families are encouraged to raise concerns with their treating clinicians within a pre-determined tiered response (Fig 1). The first step involves directly approaching the bedside staff. If the patient/family concerns remain unresolved, they are escalated to the senior ward nursing staff and doctor (step two). Beyond that, escalation is to the most senior doctor from the patient’s primary care team (step three). Consumers can also ask staff, and staff themselves can choose to, bypass any of these steps and directly activate the RRT. In such cases, response is provided by the same RRT that responds to clinician escalations. The process is only intended for clinical concerns. The system was not piloted in the local environment prior to implementation and its introduction occurred separate to our survey evaluations.

Outside of this study, staff education and awareness activities commenced three weeks prior to the introduction of the consumer escalation system. Information was delivered through in-person education sessions, network wide emails, and a non-compulsory on-line learning package. A policy document outlining required staff actions was also released. Electronic media, posters, and brochures were available for patients and family members/carers.

### Survey tool

A study specific survey was developed through a multistage process. A literature review was firstly completed to establish the current evidence surrounding consumer escalation systems in acute hospital settings [35]. Findings highlighted staff attitudes and actions that have the potential to impact upon the uptake and effectiveness of consumer escalation processes, assisted in defining key concepts, and identified deficiencies in the current evidence base requiring investigation.

A draft data collection tool was developed through a collaborative approach between members of the research team with medical, nursing, and allied health backgrounds. Questions were then reviewed by members from the local Safety and Quality Committee to ensure relevance, clarity, and face validity. As the survey was design primarily for local evaluation purposes, it did not undergo a process of formal validation. Members of the research team self-tested the ease of usability and technical functionality of the online survey. Their feedback was incorporated into the survey design prior to general release.

A series of 35 and 36 questions were presented within the first and second surveys, respectively. An additional seven questions were also available within the second survey, dependent upon a participant’s response within adaptive questions. Alterations to questions between the first and second surveys occurred to better reflect the local escalation process, including the expected actions of staff. Adaptions also occurred to capture actual experiences and to measure staff awareness levels after system introduction.

Questions were grouped into clusters to facilitate ease of survey completion. Likert scales examined staff perceptions towards the ability of patients/families to detect acute clinical deterioration and to then escalate their concerns. Likert scales also considered staff perceptions towards their responsibilities in responding to consumer concerns and the impact of introducing a local consumer escalation system. The decision to utilise a 5-point scale (from strongly disagree to strongly agree with neutral as a mid-point) was based upon prior research associated with agree-disagree scale length [38]. Surveys also included rating scales (from 0 to 100) to evaluate staff confidence in the ability and reliability of clinicians, the RDR observation chart, and consumers to identify acute clinical deterioration. Yes/no and multiple-choice questions addressed hospital staff awareness of the local consumer escalation system, response to prior situations of consumer concern, and staff demographics. Finally, a single open-ended question was provided within each survey. This explored staff understanding of consumer escalation (first survey) and acute clinical deterioration (second survey). The open question was altered between the two survey periods as concerns existed that staff may be defining clinical deterioration in a different manner to healthcare consumers. Staff were not provided with a formal definition of clinical deterioration within either survey.

The anonymous surveys were developed within the secure Research Electronic Data Capture (REDCap) tool hosted at our institution [39]. Data collection and initial management occurred through the REDCap platform [39]. The response database was stored on the Hospital’s secure username and password protected computer system with access restricted to study investigators.

Copies of the full surveys are provided within the supporting information (see S2 File).

### Survey distribution and sample

Information, reminders, and a link to the voluntary online surveys were distributed, via email, to healthcare staff across the LHN using the Network’s weekly update, and monthly Safety and Quality and Clinical Governance Bulletins. The survey distribution and sampling method was selected based on being feasible whilst allowing the capacity to reach a widespread sample.

As evaluation frameworks recognise the importance of tracking and comparing variables over time [40], including to capture change [41], the surveys were repeated at two timepoints. Both survey periods were open for approximately 15 weeks. Survey 1 (“pre”) commenced in July 2019. This period incorporated the lead up to, and initial weeks of, the introduction of the local consumer escalation system. Survey 2 (“post”) commenced in June 2020, approximately nine months after system introduction.

The final sample size for each survey was determined by the number of responses received within the specified data-collection period.

### Human research ethics committee approval

The completion of staff surveys was approved by the LHN Safety and Quality Committee and Hospital Executives as part of local evaluation and quality processes. Human Research Ethics Committee and Governance approvals were obtained (CALHN HREC Reference Number 13231; Governance Reference Number P2275) to allow the data collected through the voluntary and anonymous surveys to be analysed and used within a research project.

### Analysis

Owing to the risk of bias in rating scale measures [42, 43], all submitted surveys were screened for situations in which participants failed to utilise the full response spectrum. This occurred to help identify, for example, potential acquiescence response bias and neutral responding. Data was then analysed using descriptive statistics. Further analysis was completed within IBM SPSS Statistics version 27 with the calculations for each question being based upon the number who responded to that question. Crosstabulations and the chi-square test of independence were utilised in comparing Likert scale responses between the first and second surveys. Following preliminary analysis, it was deemed necessary to cluster Likert data from five categories down to three groups (agree, neutral and disagree). This occurred to maintain the assumption of the chi-square test of an expected count of five or more in at least 80 percent of cells [44]. In evaluating for change in staff views and knowledge following local system introduction, sub analysis was completed within the categories relating to both staff work area and role (see S3 File).

A significance level of p<0.02 was adopted owing to the completion of multiple statistical analyses with associated reductions in sample sizes. Where a p value below this threshold was identified, a z-test was completed to establish which response categories differed significantly between the two surveys.

In examining confidence rating scales, the distributions of responses were assessed for normality prior to selecting non-parametric, independent samples median tests to complete further analysis.

Responses from open-ended questions were cross-referenced with a participant’s Likert and rating scale data. Quotes that supported or negated quantitative data were identified to illustrate findings. Content analysis was also completed according to the initial steps outlined by Erlingsson and Bryiewcz [45], adapted to open-survey responses. This involved one member of the research team reading and re-reading the responses to become familiar with the data. Where required, responses were then broken down into smaller units before being inductively coded. Codes were then organised into categories. Rather than focusing upon frequencies that would potentially risk missing the important aspect of context [46], care was taken to consider both trends and pertinent points. Findings were presented visually within concept maps (available within S1–S3 Figs). Both the content analysis and mapping were completed manually within the NVivo 12 software program.

Based on literature review findings, and study questions and aims, results were grouped within three broad categories believed to have the potential to critically impact upon consumer escalation processes. Categories included (1) Staff perceptions of patient/family ability to identify clinical deterioration and escalate concerns; (2) Staff perceptions towards responding to consumer concerns; and (3) Staff awareness, design, and impact of consumer escalation systems.

## Results

A total of 304 surveys (pre: 215; post: 89) were submitted and included in data analysis. Within the pre survey group, four surveys (1.86%) were completed in the initial weeks after local system introduction. Due to the infancy of the consumer escalation system at this stage, the surveys were retained within the “pre” data analysis, as the number and timing was not considered sufficient to unduly influence results.

Following the clustering of Likert responses into the three categories noted above, data screening identified one case (0.3%) in which a participant provided the same response on all occasions. Of the participants who responded within confidence rating scales, a potential neutral response bias was observed in 8/300 (2.6%) of cases where a score of 50 was selected for each question completed.

### Participant characteristics

**Fig 2:**
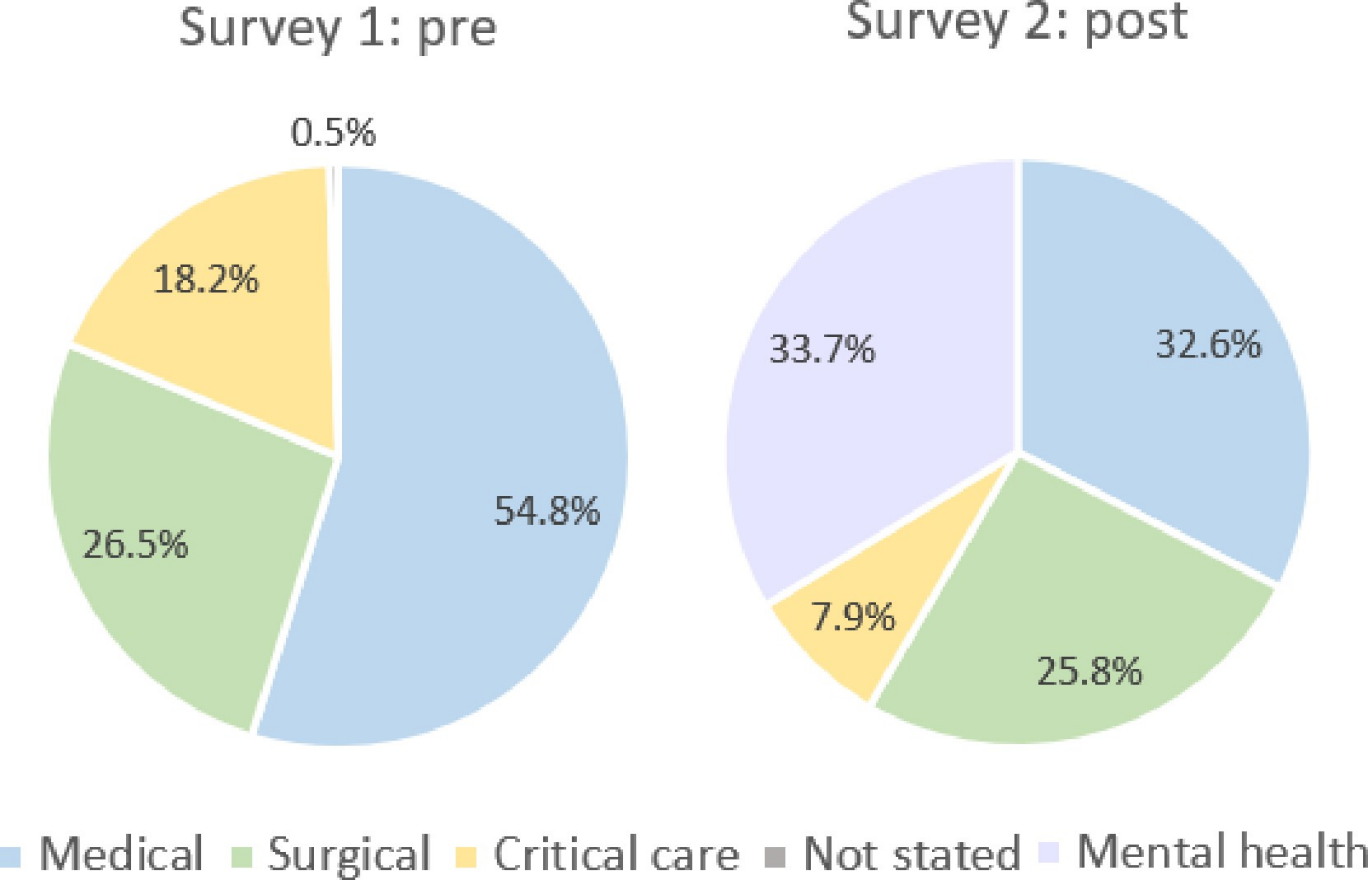
Participant area of work (%).

Within the first survey, participants included 170 (79.1%) nursing, 25 (11.6%) allied health, and 20 (9.3%) medical staff. 76 (85.4%) nurses, 8 (9%) allied health, and 5 (5.6%) medical staff completed the second survey. Experience level ranged from junior through to Consultant/senior medical staff and from junior nurse to Nursing Director. The clinical areas represented by participants are demonstrated in Fig 2. A significant difference was identified in this regard between the two survey periods (p<0.001). The mean age of respondents, where provided, was 43.3 (SD +/- 13.8) and 45.6 (SD +/- 14.3) years within the first and second surveys, respectively (p = 0.618). As only 132 of the participants provided details within the non-compulsory question of age, further analysis was not completed in this area.

### Key findings

#### Staff perceptions of patient/family ability to identify deterioration and escalate concerns

192/215 (89.3%) and 82/88 (93.2%) of pre and post survey respondents, respectively, agreed to the statement that patients and families have a sound knowledge of a patient’s normal health status and behaviour. Post implementation, there was a significant increase in the percentage of staff who agreed that healthcare consumers can be relied upon to identify changes associated with clinical deterioration (pre: 121/214 (56.5%,); post: 73/89 (82%), p <0.001) (Table 1). Sub analysis (see S3 File) demonstrated that this increase in agreement was most significant amongst nursing staff (pre: 94/169 (55.6%); post: 63/76 (82.9%), p <0.001) and within medical wards (pre: 64/118 (54.2%); post: 26/29 (89.7%), p = 0.002). Post implementation findings also suggested that staff had greater confidence in patients and families by rating them higher than allied health and junior nursing staff in their ability and reliability to identify deterioration (Table 2).

**Table 1:** Staff perceptions towards patients and family members identifying clinical deterioration and escalating concerns.

**Question/Statement**	**Survey**	**Disagree**	**Neutral**	**Agree**	**p-value**
Patients and family members have a good knowledge of a patient’s ’normal’ clinical condition and behaviour	Pre	2 (0.9%)	21 (9.8%)	192 (89.3%)	
	Post	1 (1.1%)	5 (5.7%)	82 (93.2%)	
	Total	3 (1.0%)	26 (8.6%)	274 (90.4%)	0.510
**Open survey responses from participants supporting that patients and family members have a good knowledge of a patient’s normal status:** “they often know the patients better than we do” “an important indicator of patient deterioration as the family knows the patient best” “someone who may be aware of information about the patient that clinicians are not aware of” “a valuable adjunct to nursing/medical observations”					
**Question/Statement**	**Survey**	**Disagree**	**Neutral**	**Agree**	**p-value**
Patients and family members can be relied upon to detect changes in a patient’s clinical condition indicative of acute deterioration	Pre	29 (13.6%)	64 (29.9%)	121 (56.5%)	
	Post	2 (2.2%)*	14 (15.7%)*	73 (82%)*	
	Total	31 (10.2%)	78 (25.7%)	194 (64.0%)	<0.001
**Open survey responses from participants providing a neutral response to the statement that patients and family members can be relied upon to detect changes:** “the reliability of family/friends is highly variable. The families with little experience of the health system or are anxious will be more likely to trigger an escalation event. Never the less, all escalations needs [sic] to be acted upon promptly” “They do not have the clinical, medical background to support clinical reasoning in detection [sic] a deteriorating patient. However, the concerns of loved ones should be taken into consideration as they know the patient” **Open survey responses from participants agreeing that consumers can reliably detect changes:** “family members can recognise changes in condition which may not be obvious to clinical staff before there is a change in vital observations” “nurses cannot be with the patients at all time [sic]. Family members usually are and can therefore detect changes in health” “a patient’s family members will likely know them better and be able to recognise that something is not normal [sic] than anyone who has cared for them for a short period of time”					
**Question/Statement**	**Survey**	**Disagree**	**Neutral**	**Agree**	**p-value**
Patients and family members cannot be relied upon to recognise clinical deterioration in a patient’s condition	Pre	120 (56.3%)	42 (19.7%)	51 (23.9%)	
	Post	62 (69.7%)	15 (16.8%)	12 (13.5%)	
	Total	182 (60.3%)	57 (18.9%)	63 (20.9%)	0.067
**Open survey responses from participants providing a neutral response to the statement that patients and family members cannot be relied upon to recognise clinical deterioration:** “patient and family members knowledge of medical issues will differ greatly” “not always accurate as they can be highly emotional” **Open survey responses from participants agreeing that patients and family members cannot be relied upon to recognise clinical deterioration:** “In some respects it’s important but sometimes largely inaccurate.” “I think for health literate family yes it may work but in a fair majority of cases it won’t.” “they are not objective clinicians with years of training and assessment” “asking untrained people to make a clinical judgement when they are at their most anxious and completely out of their element.”					
**Question/Statement**	**Survey**	**Disagree**	**Neutral**	**Agree**	**p-value**
Patients and family members are sufficiently confident to raise concerns about clinical deterioration in a patient’s condition with the ward staff	Pre	27 (12.7%)	74 (34.7%)	112 (52.6%)	
	Post	20 (22.7%)	20 (22.7%)	48 (54.5%)	
	Total	47 (15.6%)	94 (31.2%)	160 (53.2%)	0.031

*significant difference in proportions between pre and post survey responses

**Table 2:** Staff confidence in the ability and reliability of clinicians, the RDR chart, and patients/family members to identify acute clinical deterioration.

Confidence rating scale (0 to 100)					
**Survey 1 –Pre introduction of local consumer escalation system**		**Median (IQR)**	**Survey 2—Post introduction of local consumer escalation system**		**Median (IQR)**
	**Consultant/Specialist**	90 (80;97)		**Consultant/Specialist**	85.5 (74.3;95)
	**MO >3 PGY/Registrar**	86 (75.5;93)		**MO >3 PGY/Registrar**	82 (65;91)
	**Nurse = />4 years’ experience**	80 (70;92)		**Nurse = />4 years’ experience**	75 (60;90)
	**RDR Chart**	73 (51;87)		**RDR Chart**	74 (50;90)
	**MO </ = 3 PGY**	72 (62;83)		**MO </ = 3 PGY**	66 (50;85)
	**Allied Health Staff**	65.5 (50;75)		**Patient &/or family**	66 (50;80)
	**Nurse </ = 3 years’ experience**	65 (50;76.8)		**Allied Health Staff**	58 (50;72.5)
	**Patient &/or family**	63 (50;76)		**Nurse </ = 3 years’ experience**	56 (43.8;78)

MO = Medical Officer; PGY = post graduate year

In contrast to the above findings, open survey responses demonstrated variable staff views. The spectrum of responses included staff who supported the ability of consumers to identify clinical change, those who acknowledge variability in patient and family ability based upon individual circumstances, and staff who expressed concern that consumers lacked the medical training required to accurately identify a worsening clinical state (Table 1 and S1 Fig). Ability aside, only 160/301 (53%) of all survey respondents agreed with the statement that patients and families have sufficient confidence to raise concerns about clinical deterioration with ward staff. A shift from neutral to disagree responses was also noted from the pre to post survey periods (p = 0.031).

#### Staff perceptions towards responding to consumer concerns of deterioration

**Fig 3:**
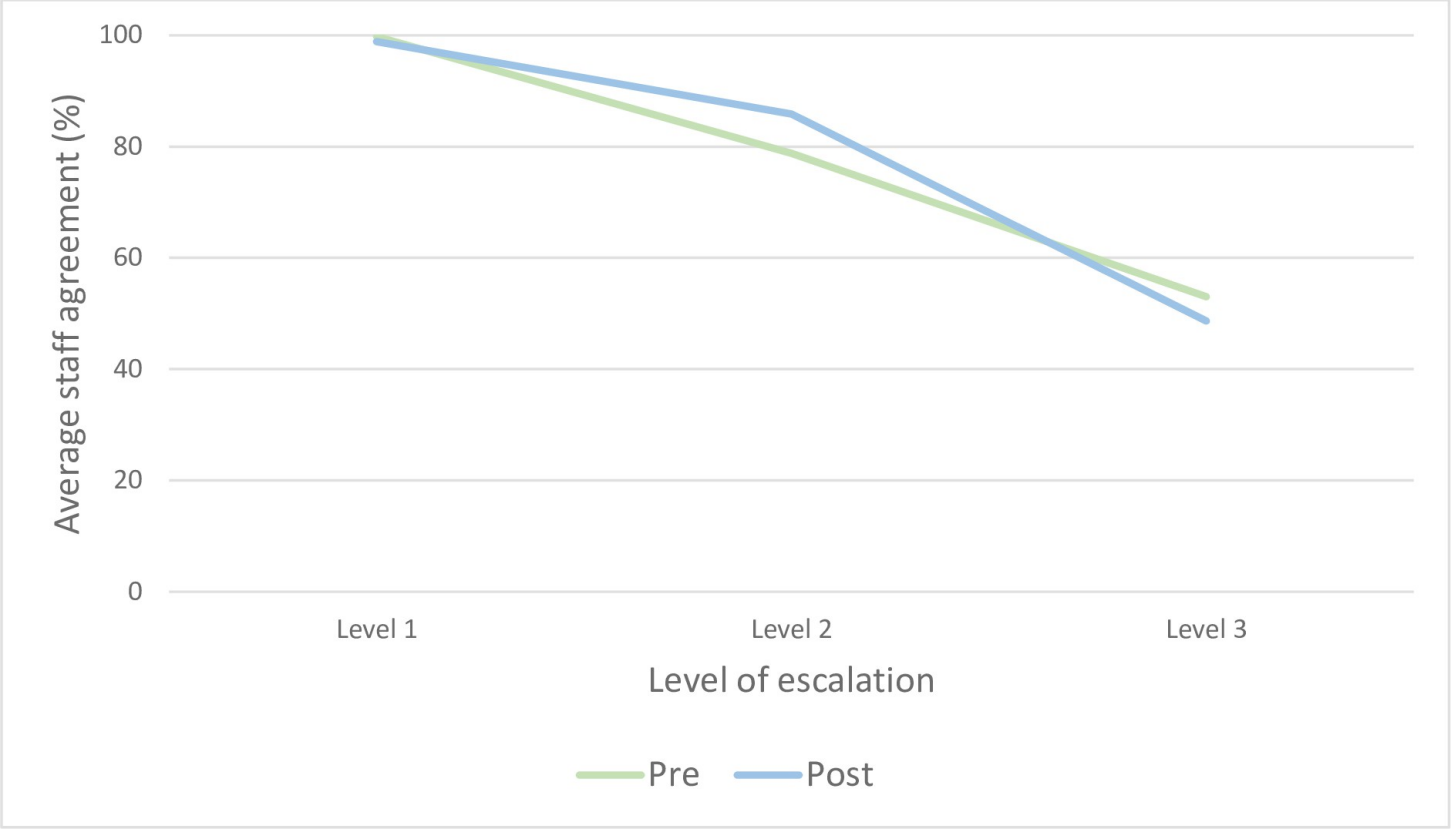
Local consumer escalation pathway: Staff agreement (%) to expected escalation requirements (steps 1 to 3).

Staff perceptions towards the actions outlined at each step in the local consumer escalation process are presented in Table 3. Within the second survey, there was an increase in the percentage of respondents agreeing that ongoing consumer concerns be escalated to a patient’s Medical Consultant/most senior medical officer (pre: 114/215 (53%); post: 60/88 (68.2%), p = 0.044), in particular amongst the nursing sample (pre: 83/170 (48.8%); post: 51/75 (68.0%), p = 0.014) (see S3 File). Despite this, aggregated data identified that, across both survey periods, staff support for the expected response requirements within the local escalation pathway decreased as the level of clinical response increased (Fig 3). Based on combined pre and post survey data, average agreement levels reduced from 99.5% at step 1, to 81.3% at step 2, and then to 50.3% at step 3.

**Table 3:** Staff perceptions towards responsibilities in response to patient and family concerns of acute clinical deterioration.

Step	Healthcare staff response	Survey	Disagree	Neutral	Agree	p-value
**1. Listen to concern and assess patient**	Listen to concerns	Pre	0 (0.0%)	0 (0.0%)	214 (100%)	
		Post	0 (0.0%)	0 (0.0%)	88 (100%)	
		Total	0 (0.0%)	0 (0.0%)	302 (100%)	-
	Assess patient, including recording vital signs	Pre	0 (0.0%)	1 (0.5%)	213 (99.5%)	
		Post	2 (2.3%)	0 (0.0%)	86 (97.7%)	
		Total	2 (0.7%)	1 (0.3%)	299 (99.0%)	0.071
**2. If concerns persist = escalate to senior staff**	Notify a senior nurse	Pre	-	-	-	
		Post	5 (5.7%)	6 (6.9%)	76 (87.4%)	
		Total	5 (5.7%)	6 (6.9%)	76 (87.4%)	-
	Notify the admitting medical team	Pre	12 (5.7%)	47 (22.2%)	153 (72.2%)	
		Post	6 (7.0%)	14 (16.3%)	66 (76.7%)	
		Total	18 (6.0%)	61 (20.5%)	219 (73.5%)	0.500
	Complete a medical or nursing review within 30 minutes	Pre	12 (5.8%)	19 (9.1%)	177 (85.1%)	
		Post	6 (7.0%)	3 (3.5%)	77 (89.5%)	
		Total	18 (6.1%)	22 (7.5%)	254 (86.4%)	0.237
	If concerns persist, the patient should be reviewed by a more senior clinician even if there is no evidence of deterioration as per the RDR chart	Pre	10 (4.7%)	34 (16.0%)	168 (79.2%)	
		Post	2 (2.3%)	7 (8.0%)	79 (89.8%)	
		Total	12 (4.0%)	41 (13.7%)	247 (82.3%)	0.094
**3. If concerns continue = escalate to Medical Consultant and RRT**	Notify the admitting Medical Consultant/most senior medical officer	Pre	-	-	-	
		Post	17 (20.7%)	31 (37.8%)	34 (41.5%)	
		Total	17 (20.7%)	31 (37.8%)	34 (41.5%)	-
	If concerns persist despite senior nurse and RMO/Registrar review, escalation should occur to the treating Consultant/most senior medical officer, even if there is no evidence of deterioration as per the RDR chart	Pre	26 (12.1%)	75 (34.9%)	114 (53.0%)	
		Post	9 (10.2%)	19 (21.6%)	60 (68.2%)	
		Total	35 (11.6%)	94 (31.0%)	174 (57.4%)	0.044
	Trigger a RRT activation	Pre	-	-	-	
		Post	17 (21.0%)	26 (32.1%)	38 (46.9%)	
		Total	17 (21.0%)	26 (32.1%)	38 (46.9%)	-
	If a patient or family member asked me to call the RRT, I would not hesitate to do so	Pre	-	-	-	
		Post	26 (29.5%)	29 (33.0%)	33 (37.5%)	
		Total	26 (29.5%)	29 (33.0%)	33 (37.5%)	-

RMO = Resident Medical Officer

48/89 (54%) of staff within the second survey indicated that they had been involved in a situation where a patient or family had expressed concern about acute clinical deterioration. In these circumstances, the most commonly reported actions related to step one of the local escalation pathway with 43/48 (89.5%) of respondents reassuring the patient and/or family member and 42/48 (87.5%) reporting increasing the frequency of patient observations. At step two, 40/48 (83%) of staff notified the admitting medical team and 30/48 (62.5%) escalated concerns to a senior nurse. Actions associated with step three occurred at the lowest frequency with 14/48 (29.2%) of staff escalating concerns to the patient’s Medical Consultant/senior medical officer and 9/48 (18.7%) activating the RRT in response to consumer concerns.

Results suggested that clinicians relied upon more than just the presence of objective indicators of deterioration in decisions about escalating clinical concerns. 248/303 (81.5%) of all pre and post respondents supported ‘worried’ (a criterion used in the absence of vital signs meeting pre-defined parameters) [47] as a valid RRT activation criteria. A similar trend was observed within negatively phrased questions whereby greater than 80% of all respondents disagreed with statements suggesting that, if there is no evidence of clinical deterioration based upon vital sign triggers, then there is no reason to escalate a clinical concern, nor is there reason for patients/families to be concerned. This finding was supported by the content analysis of open survey responses demonstrating that staff also recognised the importance of subjective considerations and a change from a patient’s baseline, in addition to objective markers, in defining clinical deterioration (see S2 Fig).

In examining clinician response to consumer concerns, consideration was also given to staff beliefs of how others may perceive them in situations of consumer escalation. Only 26/299 (8.7%) of all pre and post survey respondents acknowledged feeling apprehensive of being viewed negatively for escalating consumer concerns to a more senior clinician. 36/298 (12.1%) reported concern of being viewed negatively in a situation in which the patient/family who they were providing care to escalated concerns. No significant difference was identified between pre and post survey periods.

#### Staff awareness, design, and impact of consumer escalation systems

Pre-implementation, open responses demonstrated mixed staff attitudes towards, and variable understanding of, consumer escalation processes (see S3 Fig). Following implementation of consumer escalation, 72/89 (81%) of respondents indicated awareness of the local system.

In examining direct versus indirect consumer escalation pathways, almost all respondents agreed that patients/families should be encouraged to escalate concerns to ward staff. In contrast, only 27/212 (12.7%) of staff who responded within the first survey agreed with consumers being able to directly activate a RRT. Apprehensions about patients and families bypassing primary care clinicians, and the potential impact upon RRS resources, were identified as explanations within open responses (Table 4).

**Table 4:** Staff perceptions towards consumer escalation system pathway.

Question/Statement	Survey	Disagree	Neutral	Agree	p-value
Indirect pathway: Patients and family members should be encouraged to escalate concerns to the ward staff about clinical deterioration in a patient’s condition	Pre	1 (0.5%)	2 (0.9%)	209 (98.6%)	
	Post	2 (2.2%)	2 (2.2%)	85 (95.5%)	
	Total	3 (1%)	4 (1.3%)	294 (97.7%)	0.241
Direct pathway: Patients and family members should be able to bypass the ward staff and directly trigger a RRT if concerned about clinical deterioration in a patient’s condition	Pre	141 (66.5%)	44 (20.8%)	27 (12.7%)	
	Post	-	-	-	
	Total	141 (66.5%)	44 (20.8%)	27 (12.7%)	-
**Open survey responses from participants who disagreed with the statement that patients and family members should be able to bypass the ward staff and directly trigger a RRT:** “escalating to ward staff is appropriate for assessment” “done in consultation with the health care worker” “strong guidelines need to be in place and should not bypass senior ward staff and doctors” “the guidelines/ boundaries would have to be very clear with regards to levels of escalation and a clear demarcation as to where the family’s role in escalation ends and the physicians [sic] role takes over” “The nurses and medical staff should have the final say on the escalation of care” “needs to be controlled by clinicians”					

Post-implementation there was a significant decrease in staff concerns about increased workloads and conflict generation between patients and clinicians secondary to implementing a of a consumer escalation process. Sub analysis (S3 File) identified this change in perception to be most evident amongst nursing staff (workload pre: 73/170 (42.9%); post 11/63 (17.5%), p<0.001; staff conflict pre: 57/169 (33.7%); post 7/62 (11.3%), p = 0.003) and on medical wards (staff conflict pre: 45/116 (38.8%); post 2/23 (8.7%), p = 0.005). In contrast, a decrease in perceived positive system effects upon patient safety, patient centred care, and patient-staff rapport in association with consumer escalation was observed (Table 5). Sub analysis demonstrated a significant difference in all three of these considerations amongst allied health staff and in relation to patient-staff rapport within the nursing staff and surgical samples (see S3 File).

**Table 5:** Expected and perceived impact of consumer escalation system introduction.

Expected and perceived system impact	Survey	Disagree	Neutral	Agree	p-value
Increase workloads	Pre	67 (31.3%)	57 (26.6%)	90 (42.1%)	
	Post	40 (55.6%)*	20 (27.8%)	12 (16.7%)*	
	Total	107 (37.4%)	77 (26.9%)	102 (35.7%)	<0.001
**Open survey responses where staff responded neutral or agree to the statement that consumer escalation systems will increase workloads:** “may require additional resources” “[RRS] is extremely busy most days and this will make it more physically demanding with greater fatigue to the teams. I suspect a lot of ’boy who cried wold’ [sic] scenarios taking away the experts who may need to be at a real code when time is of the essence” “A waste of resources as the patient or family should be able to talk to a nurse and have baseline observations before [RRT] call initiated, or else those who genuinely need it may miss out by staff busy responding to "hoax" calls” “Potential for delay in responding to a real [RRT] call activated by staff because they are responding to once [sic] activated by family because they do not understand rather than true deterioration”					
**Expected and perceived system impact**	**Survey**	**Disagree**	**Neutral**	**Agree**	**p-value**
Increase patient safety	Pre	8 (3.7%)	33 (15.4%)	173 (80.8%)	
	Post	5 (6.9%)	18 (25.0%)	49 (68.1%)	
	Total	13 (4.5%)	51 (17.8%)	222 (77.6%)	0.077
**Open survey responses where staff agreed with the statement that consumer escalation systems will increase patient safety:** “valuable in providing a fast response to a sudden/acute deterioration in a patient’s condition” “essential for the safety, wellbeing and care of the patient” “providing the patient with an extra layer of safe care” “another tool to avoid the ’Swiss Cheese’ scenario where adverse medical events/conditions can be ameliorated before catastrophic complications or patient death occurs due to system inaction”					
**Expected and perceived system impact**	**Survey**	**Disagree**	**Neutral**	**Agree**	**p-value**
Promote patient centred care	Pre	7 (3.3%)	19 (8.9%)	188 (87.9%)	
	Post	7 (9.7%)*	12 (16.7%)	53 (73.6%)*	
	Total	14 (4.9%)	31 (10.8%)	241 (84.3%)	0.012
**Open survey responses where staff agreed with the statement that consumer escalation systems will increase patient centred care:** “a positive step towards more patient centred care” “A good way to make patient [sic] feel empowered and safe in the clinical environment” “will encourage discussion with family’s [sic], patients and health team as a group” “important for patient centered [sic] care, improved care and optimal outcomes”					
**Expected and perceived system impact**	**Survey**	**Disagree**	**Neutral**	**Agree**	**p-value**
Promote patient-staff rapport	Pre	11 (5.2%)	39 (18.3%)	163 (76.5%)	
	Post	9 (12.5%)*	25 (34.7%)*	38 (52.8%)*	
	Total	20 (7.0%)	64 (22.5%)	201 (70.5%)	0.001
**Open survey response where staff agreed with the statement that consumer escalation systems will promote patient-staff rapport:** “important to instill [sic] a trusting relationship between the patient, their family and the care team, that everyone has the patients [sic] best interests at the fore front of their mind”					
**Expected and perceived system impact**	**Survey**	**Disagree**	**Neutral**	**Agree**	**p-value**
Increase risk of generating patient-staff conflict	Pre	86 (40.4%)	56 (26.3%)	71 (33.3%)	
	Post	41 (57.7%)*	23 (32.4%)	7 (9.9%)*	
	Total	127 (44.7%)	79 (27.8%)	78 (27.5%)	0.001
**Open survey responses where staff responded neutral or agree to the statement that consumer escalation systems will generate patient-staff conflict:** “will inevitably place pressure on ward nursing and medical staff. This will in turn result in unnecessary unprofessional confrontation” “will undermine and overlook the professionals who are meant to have the duty of care”					
**Expected and perceived system impact**	**Survey**	**Disagree**	**Neutral**	**Agree**	**p-value**
No noticeable impact	Pre	-	-	-	
	Post	24 (34.3%)	25 (35.7%)	21 (30.0%)	
	Total	24 (34.4%)	25 (35.7%)	21 (30.0%)	-

*significant difference in proportions between the pre and post survey responses

## Discussion

Hospital staff have the potential to critically impact upon healthcare interventions [36]. We therefore completed surveys with medical, nursing, and allied health professionals across a spectrum of inpatient services to examine their understandings and perceptions in association with consumer escalation for acute clinical deterioration. To the best of our knowledge, this study is the one of the first dedicated to exploring staff views and knowledge in relation to this topic within an adult care setting.

Key study findings identified that amongst clinicians, there was variable understanding and perceptions towards consumer escalation. Staff largely agreed that patients and families have a sound knowledge of a patient’s normal health status. However, support for the ability of consumers to identify deterioration was not universal. Almost all staff agreed that patients and families should be encouraged to escalate concerns of deterioration to a ward level. Yet, this support wavered as the degree of escalation and expected clinical response increased. Whilst acknowledging that study limitations may impact on the ability to compare pre and post survey findings and to generalise results, our outcomes do not support apprehensions that consumer escalation systems increase workloads or generate conflict. However, findings do suggest that barriers may be limiting consumer escalation processes, as staff perceptions towards positive system effects decreased after local system introduction.

### Support for the ability of patients and relatives to identify clinical deterioration

Consumer escalation systems have been introduced upon coronial findings and incident investigations associated with preventable patient deaths attributed, in part, to deficiencies in staff response to patient/family concerns [13]. Whether healthcare consumers can identify deterioration on a wider scale remains uncertain. This study identified strong support amongst clinicians that adult patients/families have a sound understanding of a patient’s ‘normal’ or baseline health status and behaviours. The value of this unique insight was acknowledged, as an additional source of information to assist staff in assessing for, and identifying, deteriorating patients. This finding is consistent with those from paediatric hospitals, where clinicians supported parents’ distinctive knowledge of their child’s baseline health [21] and capability to detect early and subtle changes of clinical deterioration [24, 25].

In contrast, some staff also expressed ambivalence that this capacity may vary between individuals secondary to, for example, emotional status, health literacy level, or previous medical experiences. Staff also noted that patients/families may not have adequate confidence to raise their concerns about deterioration. Collectively, this highlights important considerations as to whether consumer escalation processes are effectively meeting the diverse needs of healthcare consumers, including those from potentially vulnerable groups. This is an area that has been largely under explored.

Study findings suggested increased staff confidence in the ability and reliability of patient and family to detect clinical deterioration within the second survey. However, based upon the current data, it is not possible to determine if this related to the introduction of a consumer escalation system or other confounding factors. A finding of potentially greater significance was that, across both survey periods, there appeared to be a “limit” to this confidence, with clinician support for responding to consumer concerns decreasing as the degree of escalation, and thus clinical concern, increased. Other research has identified that staff do not consider the early stages of responding to consumer concerns as differing significantly from their normal practice [25]. This may provide some explanation for our observations. Alternatively, some studies have suggested that consumers and clinicians may consider clinical concern [23], illness severity [21], and emergency [17] in differing manners and that because cases of consumer concern may not meet predefined objective criteria, they may be afforded less importance by staff [23]. Yet, to this last point, most clinicians in this study supported escalating their own clinical concerns, even in the absence of objective evidence. Further research is required to better understand the factors that influence the confidence that staff afford to consumer concern and patient/family understanding of, and ability to detect, clinical deterioration.

### Escalation processes for acute clinical deterioration

Hospital staff are key stakeholders in consumer escalation processes. They have roles in providing information to patients and families about consumer escalation systems, responding to consumer concerns, and creating the culture in which the system exists. This is an important consideration given that healthcare interventions, in general, risk being undermined in the longer term if there is a failure to consider the acceptability of the intervention to those who are required to deliver it [48].

Our findings indicated variability in staff attitudes towards consumer escalation systems. Some respondents labelled such processes as ‘critical’, ‘essential’, ‘imperative’ and ‘paramount’. In contrast, others expressed apprehensions, highlighting the need for clear guidelines and boundaries. Findings did, however, identify that support for patients and families to escalate concerns to ward staff was almost universal. Of note, following the implementation of consumer escalation, there was a significant increase in respondents agreeing that consumer concerns be escalated to a patient’s most senior member of medical staff. It was not possible to determine if this was the result of implementing a consumer escalation system or other factors.

Deeper reservations were noted towards patients/families bypassing staff to directly activate a RRT. Although this may reflect a degree of misunderstanding within the local setting, where an indirect model of consumer escalation was in place, other studies have also identified opposition from clinicians towards direct consumer RRT activation [21]. This has, in part, been based on staff assumptions that healthcare consumers do not have the medical training to determine when it is appropriate to make such decisions [21], and that they may make calls for inappropriate [21, 24, 28], insignificant [20], or non-emergency matters [22], thus imposing demand upon resources [21, 24] and risking RRS effectiveness [21]. To date, there has been limited evidence to support such apprehensions, with most reported cases of consumer escalation appearing justifiable based on improving patient safety or care quality [35].

Consideration needs to be given to the complexity of introducing consumer escalation within hospital environments [15, 24]. An ideal or standardised model of consumer escalation is yet to be determined. Challenges also persist in designing systems that are both supported by staff whilst still promoting collaboration and safety for patients/families if they feel that their concerns about acute deterioration have not been appropriately responded to. Advancements may emerge with research now considering a co-designed approach in the development of consumer escalation interventions [34].

### Impact of consumer escalation system introduction

Our findings did not support concerns that consumer escalation increased staff workload, or generated conflict between patients and clinicians. However, the system was under-utilised, with only six cases in which the patient/family concern about clinical deterioration was escalated to the RRT being reported during the study period. To provide context, this number represents only 0.08% of the total 7,077 RRS activations that occurred within the period between the opening of the first survey and closing of the second survey. Our findings are consistent with others showing that, despite initial staff apprehensions, there has been no evidence to date that consumer escalation systems substantially increased work demand [29, 49].

Consumer escalation systems are centred upon increasing patient safety and promoting partnership in care [13, 14]. The results of this study suggested that staff did not share this view. Instead, staff support for consumer escalation as a way of promoting patient and staff rapport, patient centred care, and patient safety decreased after system introduction. Other studies have identified reservations amongst clinicians towards consumer escalation [21, 22, 24, 28] including concerns that ward staff [28], therapeutic relationships [21, 24], oversight of care [22], professional boundaries, and staff communication will be undermined, and situations of distrust created [24]. However, such perceptions have typically responded to targeted education programs [49] to the extent that system introduction was ultimately attributed to promoting positive organisational wide cultural changes [20, 22]. The impact of staff education was not assessed in this study. Repeating surveys after a sustained period of staff education may therefore be of value.

### Local learning and potential barriers

Evaluation is essential to understanding the impact of interventions [50] and to guiding improvement processes [40, 50]. An important part of this process is considering practical aspects, including obstacles to change [50] in the real-world, clinical setting [48].

Our outcomes suggest that barriers exist. However, based on the current data, it is only possible to hypothesise as to what the barriers may be. Apprehensions, differing or negative perceptions, and limitations in the understanding and awareness of some staff in association with consumer escalation are potential examples that correspond with the outcomes of other studies [21, 23–25]. Such considerations also link with the wider literature, which has identified that aspects such as staff commitment, understanding, role identity, and confidence all have the potential to impact upon the implementation of hospital-based interventions [36].

Based upon the experiences reported in other facilities, the outcomes of such barriers have contributed to staff experiencing difficulties in educating patients and families about consumer escalation processes [17] and clinicians being selective in which patients and families they provide information to [25]. Low patient and family awareness of consumer escalation processes has in turn been associated with limited system usage or system activations for miscommunications, rather than intended purposes [20]. Barriers associated with negative staff perceptions or biases have also been identified to impact on staff response to patient/family concerns [23].

Another consideration is that the lower response rate within the second survey reported here may also reflect a decreasing staff awareness of consumer escalation processes after the initial introduction phase. Without ongoing active promotion, the concept of consumer escalation may not have the opportunity to become accepted and embedded in the hospital culture to achieve full effectiveness.

### Limitations

Our study was completed with staff working within a Local Health Network delivering health services to adult patients. Our findings may therefore not be reflective of staff views towards consumer escalation in other settings. Owing to variations in consumer escalation systems both in Australia and internationally, our findings may also not be generalisable to health services with differing consumer escalation systems.

A voluntary response sample created a risk of a sampling bias [51]. Respondents were primarily nursing staff. Whilst nurses formed almost 70% of the population from which the sample was obtained, the proportion of nursing staff in comparison to medical and allied health professionals within our sample still exceeded this value. The views of patients and family members were also not captured. The smaller sample and uneven distribution of specialities (with a focus primarily from mental health) within the second survey may have impacted upon evaluations, findings, and the generalisability of results.

Owing to the absence of a survey tool specific to the research topic, study investigators were required to develop a new instrument that was not formally validated. Several alterations were required to questions between the first and second surveys. Whilst this was considered necessary, as has been explained above, the changes did impact upon comparisons between the two survey periods. The primarily quantitative nature of survey questions may have limited the depth of findings and the ability to explain all outcomes. Despite attempts to screen data; rating scale responses do carry an inherent risk of bias. Survey questions primarily focused on staff perceptions and knowledge, and therefore, may not accurately reflect staff behaviours and actions in the real-world clinical setting. They may also not reflect actual patient and family confidence and ability to identify deterioration and escalate concerns.

Repeating surveys on two occasions assisted in the formulation of hypotheses. However, it is not possible to conclusively determine if the results observed reflected the impact of introducing a consumer escalation system, the impact of our sampling processes, or if they were the influence of another unknown factor. That noted, the authors were not aware of any additional, significant, internal or external factors that may have influenced the results between the two surveys, other than those already addressed.

Finally, our sub analysis was limited to participant’s role and work area.

## Conclusion

As a hospital-based intervention, consumer escalation systems inevitably require the support of staff and a cultural change. Within our surveys, we found that clinicians perceived patients and families to have a sound knowledge of a patient’s baseline health status and that they should be encouraged to escalate concerns of clinical deterioration at a ward level. However, beyond this, staff perceptions varied, support was not universal, and some reluctances and apprehensions were apparent.

Staff perceptions towards the positive effects of a consumer escalation system decreased over time. Consequently, consideration needs to be given to barriers that may be limiting consumer escalation processes from achieving their intended effect. Such barriers may relate to the perceptions, knowledge, and actions of hospital staff. They may also stem from other factors, including whether patients and families have the confidence and ability to raise concerns. The need for further research remains ongoing if such barriers are to be fully identified and addressed. Further consideration is also required of how to develop a model of consumer escalation that clinicians have confidence in, whilst still maintaining the underlying goals of promoting collaboration, safety, and pathways to healthcare consumers in situations of acute clinical deterioration.

## Supporting information

S1 Fig Content analysis mapping. (DOCX)

S2 Fig Content analysis mapping. (DOCX)

S3 Fig Content analysis mapping. (DOCX)

S1 File EQUATOR CHERRIES checklist. (DOCX)

S2 File Staff survey (pre and post). (DOCX)

S3 File Sub analysis output. (DOCX)
