# Supplementary material for: Acute clinical deterioration and consumer escalation: The understanding and perceptions of hospital staff
Source: PLoS One. 2022 Jun 16;17(6):e0269921. doi: 10.1371/journal.pone.0269921 (PMC9202900; doi:10.1371/journal.pone.0269921)
Supplement: S1 Fig — (DOCX) [file pone.0269921.s001.docx]

**
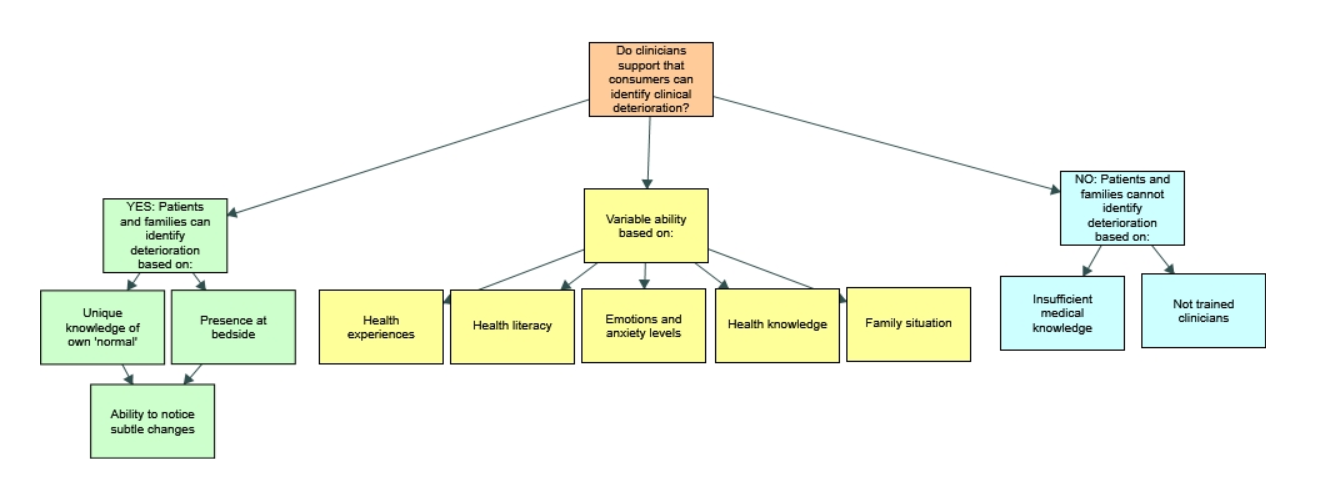
S1 Fig. Content analysis concept map: staff perceptions of patient/family ability to identify clinical deterioration**
