# Supplementary figures and images for: Acute clinical deterioration and consumer escalation: The understanding and perceptions of hospital staff
Source: PLoS One. 2022 Jun 16;17(6):e0269921. doi: 10.1371/journal.pone.0269921 (PMC9202900; doi:10.1371/journal.pone.0269921)

**
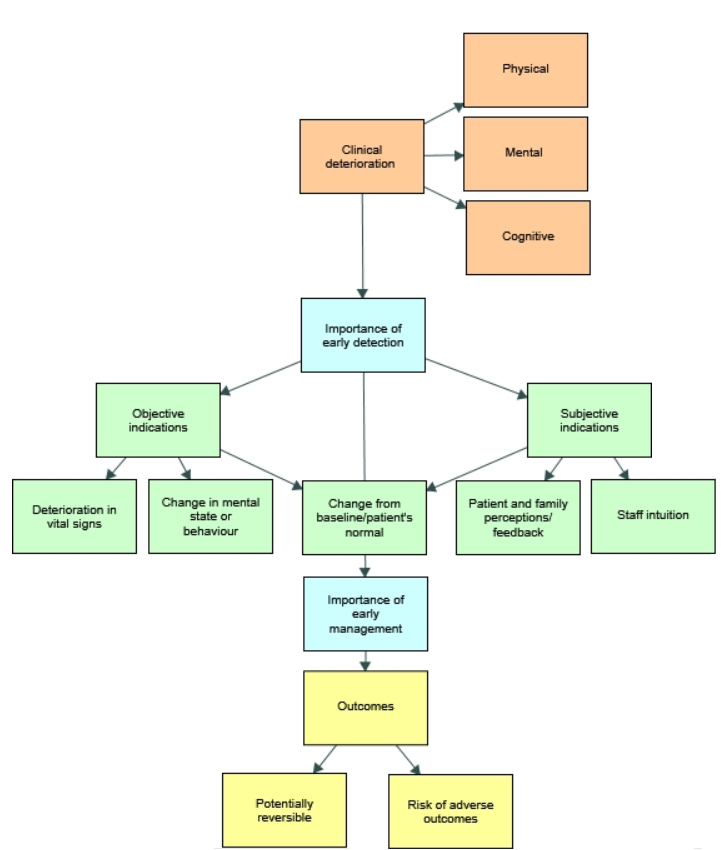
S2 Fig. Content analysis concept map: staff definitions of acute clinical deterioration**

Supplement: S2 Fig — (DOCX) [file pone.0269921.s002.docx]

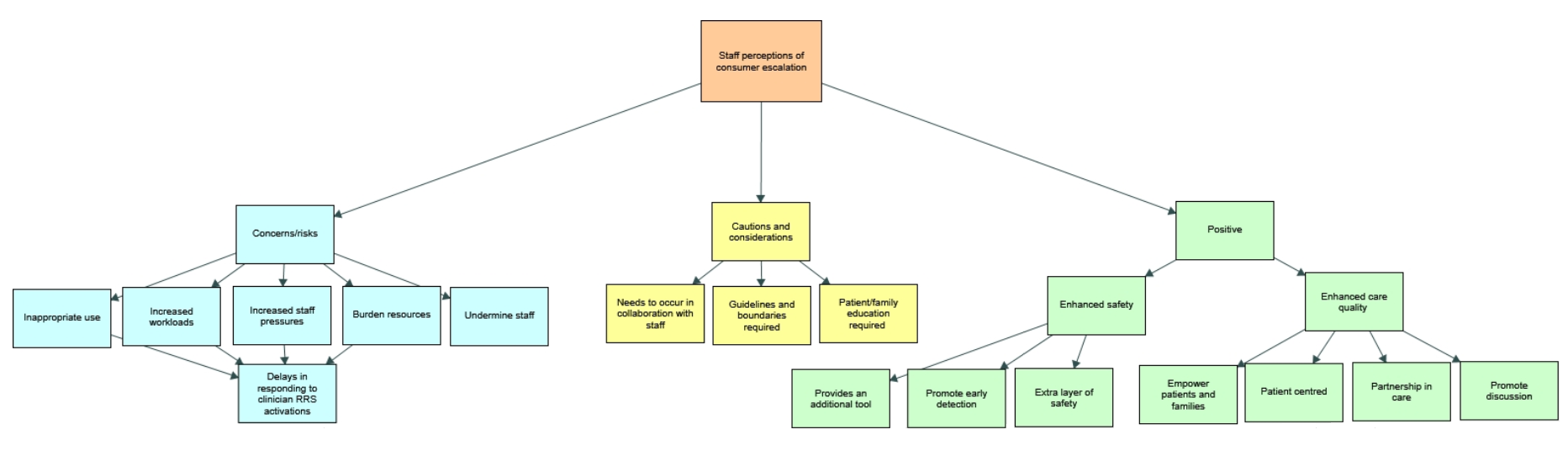
**S3 Fig. Content analysis concept map: staff perceptions of consumer escalation**

Supplement: S3 Fig — (DOCX) [file pone.0269921.s003.docx]
