# Supplementary material for: Acute clinical deterioration and consumer escalation: The understanding and perceptions of hospital staff
Source: PLoS One. 2022 Jun 16;17(6):e0269921. doi: 10.1371/journal.pone.0269921 (PMC9202900; doi:10.1371/journal.pone.0269921)
Supplement: S1 File — (DOCX) [file pone.0269921.s004.docx]

| **Item category** | **Checklist item** | **Page no.** | **Description** |
| --- | --- | --- | --- |
| Design | Survey design | 6  6  6,10 | - The target population included healthcare staff (medical, nursing, and allied health) providing care to adult hospital inpatients - The sample was derived from staff working within an Australian Local Health Network (LHN) including five hospital sites - A voluntary response sample was adopted |
| Institutional Review Board approval and informed consent processes | IRB approval | 11  11 | - Surveys were approved for distribution by the LHN Safety and Quality Committee and Hospital Executives as part of quality assurance processes - Human Research Ethics Committee and Governance approvals were obtained (CALHN HREC Reference Number 13231; Governance Reference Number P2275) to allow the data collected through the voluntary and anonymous surveys to be analysed and used within a research project |
|  | Informed consent | 6,11 | - Written consent was not obtained as all surveys were completed on an anonymous and voluntary basis.  HREC approval was obtained to permit the analysis and use of the survey data |
|  | Data protection | 10  6,10  10 | - The survey was developed within the secure REDCap application - All responses were anonymous - The response database was stored on the Hospital’s secure username and password protected computer system with access restricted to study investigators |
| Development and pre-testing | Development and testing | 8,9 | A study specific survey instrument was developed through a multistage process:   - A literature review was completed to establish current evidence and areas requiring further investigation - Collaborative drafting of questions by members of the research team - Review of questions were reviewed by the LHN Safety and Quality Committee and Hospital Executives - Modification of questions based upon collective feedback - Creation of online survey within REDCap - Members of the research team self-tested the online survey usability and technical functionality - Feedback incorporated into final survey design - Survey release |
| Recruitment process and description of the sample having access to the questionnaire | Open versus closed survey | 10 | - The survey was open to all medical, nursing, and allied health staff employed within the LHN |
|  | Contact mode | 10 | - An invitation to complete each survey was distributed, via email, across the LHN |
|  | Advertising the survey | 10 | - Information, reminders, and link to the voluntary online survey were distributed, via email, to healthcare staff across the LHN using the Network’s weekly update, and monthly Safety and Quality and Clinical Governance Bulletins |
| Survey administration | Web/E-mail | 10  10 | - The survey was accessible via a link within staff emails - Responses were automatically captured within the REDCap survey database |
|  | Context | 10 | - The survey was distributed via email rather than a website. Links to the survey were contained within emails providing staff updates in association with safety, quality, organisational governance, and other emerging clinical and non-clinical matters |
|  | Mandatory/voluntary | 6,10 | - The survey was voluntary |
|  | Incentives | NA | - No incentives were offered for completing the survey |
|  | Time/date | 10 | - The two survey periods each spanned for approximately 15 weeks. Surveys 1 (“pre”) commenced in July 2019. This period incorporated the lead up to, and initial weeks of, the introduction of the local consumer escalation system. Survey 2 (“post”) commenced in June 2020, approximately nine months after system introduction. |
|  | Randomisation of items or questions | NA | - There was no randomisation of items |
|  | Adaptive questioning | 9 | - Adaptive questioning was used on two occasion within the second survey |
|  | Number of items | 9 | - Survey 1: 35 individual items - Survey 2: 36 individual items. A further 7 items were displayed if a ‘yes’ response was provided within adaptive questions |
|  | Number of screens | - | - One screen (on which the participant was able to scroll up or down) was used for each survey |
|  | Completeness check | - | - Respondent role and area of work were mandatory items. Participants were otherwise permitted to leave items unanswered |
|  | Review step | - | - Respondents were able to review and alter their responses at any time prior to submitting the survey |
| Response rates | Unique site visitor | NA | - Not determined as the survey was distributed via email |
|  | View rate | NA | - Not applicable as the survey invitation was distributed via email |
|  | Participation rate | - | - All submitted surveys were included. |
|  | Completion rate | 13 | - All submitted surveys were counted |
| Preventing multiple entries from the same individual | Cookies used | NA | - Cookies were not used |
|  | IP check | NA | - The survey was anonymous and IP addresses were not recorded |
|  | Log file analysis | NA | - Log files were not used |
|  | Registration (closed surveys) | NA | - The survey was open to all medical, nursing, and allied health staff across the LHN. Participants were not required to register or login to access the survey |
| Analysis | Handling of incomplete questionnaires | 11 | - All submitted surveys were included in data analysis. Statistical calculations for each question were based upon the number who responded to that question |
|  | Questionnaires submitted with an atypical timestamp | NA | - Only survey commencement time was timestamped. All submitted responses were included in data analysis |
|  | Statistical correction | NA | - Weighing of items and propensity scores were not adopted in data analysis |

* Eysenbach G. Improving the quality of Web surveys: The Checklist for Reporting Results of Internet E-Surveys (CHERRIES). J Med Internet Res 2004;6:e34 [cited 4 June 2021]. Available from: <https://www.jmir.org/2004/3/e34/>.
