# Supplementary material for: Acute clinical deterioration and consumer escalation: The understanding and perceptions of hospital staff
Source: PLoS One. 2022 Jun 16;17(6):e0269921. doi: 10.1371/journal.pone.0269921 (PMC9202900; doi:10.1371/journal.pone.0269921)
Supplement: S2 File — (DOCX) [file pone.0269921.s005.docx]

**Pre-implementation Consumer Escalation – Staff Survey**

| Please indicate your role  *must provide value |  | | | | |
| --- | --- | --- | --- | --- | --- |
| Age |  | | | | |
| Please indicate the main area in which you work  *must provide value |  | | | | |
|  | | | | | |
|  | Strongly disagree | Disagree | Neutral | Agree | Strongly agree |
| If a patient or family member expresses concerns that a patient’s condition is deteriorating, healthcare staff should:   - Assess patient, including recording vital signs - Review within 30 minutes - Call the admitting medical team |  |  |  |  |  |
|  | | | | | |
|  | Strongly disagree | Disagree | Neutral | Agree | Strongly agree |
| Patients and family members have a good knowledge of a patient’s ‘normal’ clinical condition and behaviour |  |  |  |  |  |
| Patients and family members can be relied upon to detect changes in a patient’s clinical condition indicative of acute deterioration |  |  |  |  |  |
| If a patient or family member expresses concerns about deterioration in a patient's clinical condition, healthcare staff have a responsibility to listen |  |  |  |  |  |
| If a patient or family member's concerns about clinical deterioration persist, the patient should be reviewed by a more senior clinician even if there is no evidence of deterioration as per the RDR chart |  |  |  |  |  |
| If a patient or family member continued to express concerns despite review by a senior nurse and RMO/Registrar, the patient or family member's concerns should be escalated to the treating Consultant, even if there is no evidence of deterioration as per the RDR chart |  |  |  |  |  |
|  | | | | | |
|  | Strongly disagree | Disagree | Neutral | Agree | Strongly agree |
| Staff concern, or 'Worried' is a valid RRT calling criteria |  |  |  |  |  |
| You have no hesitation to trigger a RRT call if you were "Worried" |  |  |  |  |  |
| If there is no evidence of clinical deterioration as per the RDR chart, then there is no reason to escalate a clinical concern |  |  |  |  |  |
| If there is no evidence of clinical deterioration as per the RDR chart, then there is no reason for patients and family members to be concerned |  |  |  |  |  |
|  | | | | | |
|  | Strongly disagree | Disagree | Neutral | Agree | Strongly agree |
| Patients and family members should be encouraged to escalate concerns to the ward staff about clinical deterioration in a patient's condition |  |  |  |  |  |
| Patients and family members are sufficiently confident to raise concerns about clinical deterioration in a patient's condition with the ward staff |  |  |  |  |  |
| Patients and family members cannot be relied upon to recognise clinical deterioration in a patient's condition |  |  |  |  |  |
|  | | | | | |
|  | Strongly disagree | Disagree | Neutral | Agree | Strongly agree |
| Introducing systems that allow patients and family members to escalate concerns about clinical deterioration will:   - result in increased ward staff workload - make patients safer - highlight and promote patient centred care - promote patient - staff rapport - risk generating patient - staff conflict |  |  |  |  |  |
|  | | | | | |
|  | Strongly disagree | Disagree | Neutral | Agree | Strongly agree |
| I am concerned that I will be viewed negatively if patients or family member for whom I am providing care escalates concerns about clinical deterioration |  |  |  |  |  |
| I am concerned that I will be viewed negatively if I escalated a patient’s or family member's concerns about clinical deterioration with my more senior staff (e.g. a senior nurse or more senior medical officer) |  |  |  |  |  |
| Patients and family members should be able to bypass the ward staff and directly trigger a RRT call if concerned about clinical deterioration in a patient's condition |  |  |  |  |  |
|  | | | | | |
| On the following scale please rate the following with respect to your confidence in their utility/reliability to detect clinical deterioration:   - Rapid Detection and Response Adult Observation Chart - Nurse (< /= 3 years’ experience) - Nurse (>/= 4 years’ experience) - Allied Health staff - Doctor (< /= PGY3) - Doctor (Registrar level) - Doctor, Consultant level - Patients and/or family members | Sliding scale 0 to 100:  0 (Lowest) ________ (50) Neutral ________100 (Highest) | | | | |
|  | | | | | |
| Please complete the following sentence. "Patient and Family/Carer Activated Escalation of Care" is… |  | | | | |
| **Submit** | | | | | |

**Post-implementation Consumer Escalation – Staff Survey**

| Please indicate your role  *must provide value |  | | | | |
| --- | --- | --- | --- | --- | --- |
| Age |  | | | | |
| Please indicate the main location in which you work |  | | | | |
| Please indicate the main Program in which you work  *must provide value |  | | | | |
|  | | | | | |
|  | Strongly disagree | Disagree | Neutral | Agree | Strongly agree |
| If a patient or family member expresses concerns that a patient’s condition is deteriorating, healthcare staff should:   - Assess patient, including recording vital signs - Notify a more senior nurse - Notify the admitting medical team - Notify the admitting medical Consultant - Medical or nurse review within 30 minutes - Trigger a RRT call |  |  |  |  |  |
|  | | | | | |
|  | Strongly disagree | Disagree | Neutral | Agree | Strongly agree |
| Patients and family members have a good knowledge of a patient’s ‘normal’ clinical condition and behaviour |  |  |  |  |  |
| Patients and family members can be relied upon to detect changes in a patient’s clinical condition indicative of acute deterioration |  |  |  |  |  |
| If a patient or family member expresses concerns about deterioration in a patient's clinical condition, healthcare staff have a responsibility to listen |  |  |  |  |  |
| If a patient or family member's concerns about clinical deterioration persist, the patient should be reviewed by a more senior clinician even if there is no evidence of deterioration as per the RDR chart |  |  |  |  |  |
| If a patient or family member continued to express concerns despite review by a senior nurse and RMO/Registrar, the patient or family member's concerns should be escalated to the treating Consultant, even if there is no evidence of deterioration as per the RDR chart |  |  |  |  |  |
|  | | | | | |
|  | Strongly disagree | Disagree | Neutral | Agree | Strongly agree |
| Staff concern, or 'Worried' is a valid RRT calling criteria |  |  |  |  |  |
| You have no hesitation to trigger a RRT call if you were "Worried" |  |  |  |  |  |
| If there is no evidence of clinical deterioration as per the RDR chart, then there is no reason to escalate a clinical concern |  |  |  |  |  |
| If there is no evidence of clinical deterioration as per the RDR chart, then there is no reason for patients and family members to be concerned |  |  |  |  |  |
|  | | | | | |
|  | Strongly disagree | Disagree | Neutral | Agree | Strongly agree |
| Patients and family members should be encouraged to escalate concerns to the ward staff about clinical deterioration in a patient's condition |  |  |  |  |  |
| Patients and family members are sufficiently confident to raise concerns about clinical deterioration in a patient's condition with the ward staff |  |  |  |  |  |
| Patients and family members cannot be relied upon to recognise clinical deterioration in a patient's condition |  |  |  |  |  |
|  | | | | | |
| **I am aware that our hospital network has a Consumer Escalation process called “You’re Worried, We’re Listening”^#^** | Yes  No | | | | |
|  | Strongly disagree | Disagree | Neutral | Agree | Strongly agree |
| **Since the introduction of the ‘You’re Worried, We’re Listening’ Consumer Escalation initiative across our hospital network, it has had the following effect…^#^**   - **increased ward staff workload** - **made patients safer** - **highlighted and promoted patient centred care** - **promoted patient - staff rapport** - **increased risk of generating patient - staff conflict** - **had no noticeable impact** |  |  |  |  |  |
|  | | | | | |
|  | Strongly disagree | Disagree | Neutral | Agree | Strongly agree |
| I am concerned that I will be viewed negatively if patients or family member for whom I am providing care escalates concerns about clinical deterioration |  |  |  |  |  |
| I am concerned that I will be viewed negatively if I escalated a patient’s or family member's concerns about clinical deterioration with my more senior staff (e.g. a senior nurse or more senior medical officer) |  |  |  |  |  |
| If a patient or family member asked me to trigger a RRT call, I would not hesitate to do so |  |  |  |  |  |
|  | | | | | |
| **Have you been involved in a situation where a patient and/or family member has expressed to you their concerns with regard to acute clinical deterioration?^#^** | Yes  No | | | | |
| **As a consequence of the patient and/or family member raising those concerns you did which of the following… (can select more than one)^#^** | - Reassured the patient and/or family member - Increased frequency of patient observations - Notified a more senior nurse - Notified the admitting medical team - Notified the admitting medical Consultant - Triggered a RRT call | | | | |
|  | | | | | |
| On the following scale please rate your confidence in the ability/reliability of the … to detect clinical deterioration:   - Rapid Detection and Response Adult Observation Chart/criteria - Nurse (< /= 3 years’ experience) - Nurse (>/= 4 years’ experience) - Allied Health staff - Doctor (< /= 2 years post Internship) - Doctor (>/=3 years post Internship) - Specialist Doctor - Patients and/or family members | Sliding scale 0 to 100:  0 (Not at all confident) _____50(Confident) _____100 (Extremely confident) | | | | |
|  | | | | | |
| Please complete the following sentence. "I consider acute clinical deterioration to be…” |  | | | | |
| **Submit** | | | | | |

^#^Adaptive question

**NB: Questions in bold are directed specifically to examining staff experiences within the local setting.** Non-bold questions are directed at examining staff perceptions towards consumer escalation systems in general.
