# Supplementary material for: Acute clinical deterioration and consumer escalation: The understanding and perceptions of hospital staff
Source: PLoS One. 2022 Jun 16;17(6):e0269921. doi: 10.1371/journal.pone.0269921 (PMC9202900; doi:10.1371/journal.pone.0269921)
Supplement: S3 File — (DOCX) [file pone.0269921.s006.docx]

| **Pre and post agreement levels: Participant role** | | | | | | | |
| --- | --- | --- | --- | --- | --- | --- | --- |
| **Question/statement** |  | **Medical** | **p value** | **Nursing** | **p value** | **Allied Health** | **p value** |
| **Patients and family members have a good knowledge of a patient's 'normal' clinical condition and behaviour** | Pre | Agree 14/20 (70.0%)  Neutral 6/20 (30.0%)  Disagree 0/20 (0%) | .160 | Agree 155/170 (91.2%)  Neutral 13/170 (7.6%)  Disagree 2/170 (1.2%) | .960 | Agree 23/25 (92.0%)  Neutral 2/25 (8.0%)  Disagree 0/25 (0%) | .409 |
|  | Post | Agree 5/5 (100%)  Neutral 0/5 (0%)  Disagree 0/5 (0%) |  | Agree 69/75 (92.0%)  Neutral 5/75 (6.7%)  Disagree 1/75 (1.3%) |  | Agree 8/8 (100%)  Neutral 0/8 (0%)  Disagree 0/8 (0%) |  |
| **Patients and family members can be relied upon to detect changes in a patient's clinical condition indicative of acute deterioration** | Pre | Agree 7/20 (35.0%)  Neutral 10/20 (50.0%)  Disagree 3/20 (15.0%) | .182 | Agree 94/169 (55.6%)  Neutral 52/169 (30.8%)  Disagree 23/169 (13.6%) | <.001 | Agree 20/25 (80.0%)  Neutral 2/25 (8.0%)  Disagree 3/25 (12.0%) | .298 |
|  | Post | Agree 4/5 (80.0%)  Neutral 1/5 (20.0%)  Disagree 0/5 (0%) |  | Agree 63/76 (82.9%)  Neutral 11/76 (14.5%)  Disagree 2/76 (2.6%) |  | Agree 6/8 (75.0%)  Neutral 2/8 (25.0%)  Disagree 0/8 (0%) |  |
| **Patients and family members cannot be relied upon to recognise clinical deterioration in a patient's condition** | Pre | Agree 8/20 (40.0%)  Neutral 3/20 (15.0%)  Disagree 9/20 (45.0%) | .707 | Agree 35/169 (20.7%)  Neutral 39/169 (23.1%)  Disagree 95/169 (56.2%) | .129 | Agree 8/24 (33.3%)  Neutral 0/24 (0%)  Disagree 16/24 (66.7%) | .136 |
|  | Post | Agree 1/5 (20.0%)  Neutral 1/5 (20.0%)  Disagree 3/5 (60.0%) |  | Agree 10/76 (13.2%)  Neutral 13/76 (17.1%)  Disagree 53/76 (69.7%) |  | Agree 1/8 (12.5%)  Neutral 1/8 (12.5%)  Disagree 6/8 (75.0%) |  |
| **Patients and family members are sufficiently confident to raise concerns about clinical deterioration in a patient's condition with the ward staff** | Pre | Agree 5/20 (25.0%)  Neutral 11/20 (55.0%)  Disagree 4/20 (20.0%) | .366 | Agree 99/169 (58.6%)  Neutral 53/169 (31.4%)  Disagree 17/169 (10.1%) | .163 | Agree 8/24 (33.3%)  Neutral 10/24 (41.7%)  Disagree 6/24 (25.0%) | .148 |
|  | Post | Agree 2/5 (40.0%)  Neutral 1/5 (20.0%)  Disagree 2/5 (40.0%) |  | Agree 45/75 (60.0%)  Neutral 17/75 (22.7%)  Disagree 13/75 (17.3%) |  | Agree 1/8 (12.5%)  Neutral 2/8 (25.0%)  Disagree 5/8 (62.5%) |  |
| **If patient/family express concerns about deterioration in a patient’s condition, healthcare staff have a responsibility to listen** | Pre | Agree 20/20 (100%)  Neutral 0/20 (0%)  Disagree 0/20 (0%) | NA | Agree 170/170 (100%)  Neutral 0/170 (0%)  Disagree 0/170 (0%) | NA | Agree 24/24 (100%)  Neutral 0/24 (0%)  Disagree 0/24 (0%) | NA |
|  | Post | Agree 5/5 (100%)  Neutral 0/5 (0%)  Disagree 0/5 (0%) |  | Agree 75/75 (100%)  Neutral 0/75 (0%)  Disagree 0/75 (0%) |  | Agree 8/8 (100%)  Neutral 0/8 (0%)  Disagree 0/8 (0%) |  |
| **If a patient or family member expresses concerns that a patient’s condition is deteriorating, healthcare staff should: Assess patient, including recording vital signs** | Pre | Agree 20/20 (100%)  Neutral 0/20 (0%)  Disagree 0/20 (0%) |  | Agree 169/170 (99.4%)  Neutral 1/170 (0.5%)  Disagree 0/170 (0%) |  | Agree 24/24 (100%)  Neutral 0/24 (0%)  Disagree 0/24 (0%) |  |
|  | Post | Agree 5/5 (100%)  Neutral 0/5 (0%)  Disagree 0/5 (0%) | NA | Agree 74/76 (97.4%)  Neutral 0/76 (0%)  Disagree 2/76 (2.6%) | .084 | Agree 7/7 (100%)  Neutral 0/7 (0%)  Disagree 0/7 (0%) | NA |
| **If a patient or family member expresses concerns that a patient’s condition is deteriorating, healthcare staff should: Notify a senior nurse** | Pre |  |  |  |  |  |  |
|  | Post | Agree 4/5 (80.0%)  Neutral 0/5 (0%)  Disagree 1/5 (20.0%) |  | Agree 67/75 (89.3%)  Neutral 5/75 (6.7%)  Disagree 3/75 (4.0%) |  | Agree 5/7/ (71.4%)  Neutral 1/7 (14.3%)  Disagree 1/7 (14.3%) |  |
| **If a patient or family member expresses concerns that a patient’s condition is deteriorating, healthcare staff should: Notify the admitting medical team** | Pre | Agree 14/20 (70.0%)  Neutral 5/20 (25.0%)  Disagree 1/20 (5.0%) | .373 | Agree 118/167 (70.7%)  Neutral 39/167 (23.4%)  Disagree 10/167 (6.0%) | .431 | Agree 21/25 (84.0%)  Neutral 3/25 (12.0%)  Disagree 1/25 (4.0%) | .193 |
|  | Post | Agree 5/5 (100%)  Neutral 0/5 (0%)  Disagree 0/5 (0%) |  | Agree 58/75 (77.3%)  Neutral 12/75 (16.0%)  Disagree 5/75 (6.7%) |  | Agree 3/6 (50.0%)  Neutral 2/6 (33.3%)  Disagree 1/6 (16.7%) |  |
| **If a patient or family member expresses concerns that a patient’s condition is deteriorating, healthcare staff should: Complete a medical or nursing review within 30 minutes** | Pre | Agree 16/20 (80.0%)  Neutral 4/20 (20.0%)  Disagree 0/20 (0%) | .275 | Agree 143/166 (86.1%)  Neutral 13/166 (7.8%)  Disagree 10/166 (6.0%) | .521 | Agree 18/22 (81.8%)  Neutral 2/22 (9.1%)  Disagree 2/22 (9.1%) | .269 |
|  | Post | Agree 5/5 (100%)  Neutral 0/5 (0%)  Disagree 0/5 (0%) |  | Agree 68/75 (90.7%)  Neutral 3/75/ (4.0%)  Disagree 4/75 (5.3%) |  | Agree 4/6 (66.7%)  Neutral 0/6 (0%)  Disagree 2/6 (33.3%) |  |
| **If patient/family concerns about clinical deterioration persist, the patient should be reviewed by a more senior clinician even if there is no evidence of deterioration as per the RDR chart** | Pre | Agree 18/20 (90.0%)  Neutral 1/20 (5.0%)  Disagree 1/20 (5.0%) | .492 | Agree 129/167 (77.2%)  Neutral 30/167 (18.0%)  Disagree 8/167 (4.8%) | .020 | Agree 21/25 (84.0%)  Neutral 3/25 (12.0%)  Disagree 1/25 (4.0%) | .590 |
|  | Post | Agree 4/5 (80.0%)  Neutral 1/5 (20.0%)  Disagree 0/5 (0%) |  | Agree 69/75 (92.0%)  Neutral 4/75 (5.3%)  Disagree 2/75 (2.7%) |  | Agree 6/8 (75.0%)  Neutral 2/8 (25.0%)  Disagree 0/8 (0%) |  |
| **If a patient or family member expresses concerns that a patient’s condition is deteriorating, healthcare staff should: Notify the admitting Medical Consultant/most senior medical officer** | Pre |  |  |  |  |  |  |
|  | Post | Agree 4/5 (80.0%)  Neutral 1/5 (20.0%)  Disagree 0/5 (0%) |  | Agree 28/72 (38.9%)  Neutral 27/72 (37.5%)  Disagree 17/72 (23.6%) |  | Agree 2/5 (40.0%)  Neutral 3/5 (60.0%)  Disagree 0/5 (0%) |  |
| **If patient/family concerns about clinical deterioration persist despite senior nurse and RMO/Registrar review, escalation should occur to the treating Consultant/most senior medical officer, even if there is no evidence of deterioration as per the RDR chart** | Pre | Agree 14/20 (70.0%)  Neutral 2/20 (10.0%)  Disagree 4/20 (20.0%) |  | Agree 83/170 (48.8%)  Neutral 67/170 (39.4%)  Disagree 20/170 (11.8%) |  | Agree 17/25 (68.0%)  Neutral 6/25 (24.0%)  Disagree 2/25 (8.0%) |  |
|  | Post | Agree 3/5 (60.0%)  Neutral 2/5 (40.0%)  Disagree 0/5 (0%) | .191 | Agree 51/75 (68.0%)  Neutral 16/75 (21.3%)  Disagree 8/75 (10.7%) | .014 | Agree 6/8 (75.0%)  Neutral 1/8 (12.5%)  Disagree 1/8 (12.5%) | .757 |
| **If a patient or family member expresses concerns that a patient’s condition is deteriorating, healthcare staff should: Trigger a RRT activation** | Pre |  |  |  |  |  |  |
|  | Post | Agree 0/4 (0%)  Neutral 2/4 (50.0%)  Disagree 2/4 (50.0%) |  | Agree 37/72 (51.4%)  Neutral 21/72 (29.2%)  Disagree 14/72 (19.4%) |  | Agree 1/5 (20.0%)  Neutral 3/5 (60.0%)  Disagree 1/5 (20.0%) |  |
| **If a patient or family member asked me to call the RRT, I would not hesitate to do so** | Pre |  |  |  |  |  |  |
|  | Post | Agree 0/4 (0%)  Neutral 3/4 (75.0%)  Disagree 1/4 (25.0%) |  | Agree 30/76 (39.5%)  Neutral 24/76 (31.6%)  Disagree 22/76 (28.9%) |  | Agree 3/8 (37.5%)  Neutral 2/8 (25.0%)  Disagree 3/8 (37.5%) |  |
| **Patients and family members should be encouraged to escalate concerns to the ward staff about clinical deterioration in a patient's condition** | Pre | Agree 19/20 (95.0%)  Neutral 1/20 (5.0%)  Disagree 0/20 (%) | .610 | Agree 166/168 (98.8%)  Neutral 1/168 (0.6%)  Disagree 1/168 (0.6%) | .164 | Agree 24/24 (100%)  Neutral 0/24 (0%)  Disagree 0/24 (0%) | NA |
|  | Post | Agree 5/5 (100%)  Neutral 0/5 (0%)  Disagree 0/5 (0%) |  | Agree 72/76 (94.7%)  Neutral 2/76 (2.6%)  Disagree 2/76 (2.6%) |  | Agree 8/8 (100%)  Neutral 0/8 (0%)  Disagree 0/8 (0%) |  |
| **Patients and family members should be able to bypass the ward staff and directly trigger a RRT if concerned about clinical deterioration in a patient's condition** | Pre | Agree 1/20 (5.0%)  Neutral 3/20 (15.0%)  Disagree 16/20 (80.0%) |  | Agree 23/168 (13.7%)  Neutral 34/168 (20.2%)  Disagree 111/168 (66.1%) |  | Agree 3/24 (12.5%)  Neutral 7/24 (29.2%)  Disagree 14/24 (58.3%) |  |
|  | Post |  |  |  |  |  |  |
| **Staff concern, or ‘worried’ is a valid RRT calling criteria** | Pre | Agree 15/20 (75.0%)  Neutral 4/20 (20.0%)  Disagree 1/20 (5.0%) | .285 | Agree 144/169 (85.2%)  Neutral 13/169 (7.7%)  Disagree 12/169 (7.1%) | .394 | Agree 19/25 (76.0%)  Neutral 3/25 (12.0%)  Disagree 3/25 (12.0%) | .379 |
|  | Post | Agree 2/5 (40.0%)  Neutral 2/5 (40.0%)  Disagree 1/5 (20.0%) |  | Agree 64/76 (84.2%)  Neutral 9/76 (11.8%)  Disagree 3/76 (3.9%) |  | Agree 4/8 (50.0%)  Neutral 2/8 (25.0%)  Disagree 2/8 (25.0%) |  |
| **You have no hesitations to trigger a RRT call if you are ‘worried’** | Pre | Agree 12/20 (60.0%)  Neutral 7/20 (35.0%)  Disagree 1/20 (5.0%) | .098 | Agree 136/168 (81.0%)  Neutral 14/168 (8.3%)  Disagree 18/168 (10.7%) | .063 | Agree 13/24 (54.2%)  Neutral 7/24 (29.2%)  Disagree 4/24 (16.7%) | .391 |
|  | Post | Agree 2/5 (40.0%)  Neutral 1/5 (20.0%)  Disagree 2/5 (40.0%) |  | Agree 61/76 (80.3%)  Neutral 12/76 (15.8%)  Disagree 3/76 (3.9%) |  | Agree 4/8 (50.0%)  Neutral 1/8 (12.5%)  Disagree 3/8 (37.5%) |  |
| **If there is no evidence of deterioration as per the RDR chart, then there is no reason to escalate clinical concern** | Pre | Agree 0/20 (0%)  Neutral 3/20 (15.0%)  Disagree 17/20 (85.0%) | .112 | Agree 12/166 (7.2%)  Neutral 15/166 (9.0%)  Disagree 139/166 (83.7%) | .617 | Agree 2/25 (8.0%)  Neutral 8/25 (32.0%)  Disagree 15/25 (60.0%) | .550 |
|  | Post | Agree 1/5 (20.0%)  Neutral 1/5 (20.0%)  Disagree 3/5 (60.0%) |  | Agree 5/76 (6.6%)  Neutral 10/76 (13.2%)  Disagree 61/76 (80.3%) |  | Agree 1/8 (12.5%)  Neutral 1/8 (12.5%)  Disagree 6/8 (75.0%) |  |
| **If there is no evidence of deterioration as per the RDR chart, then there is no reason for patients/family to be concerned** | Pre | Agree 1/20 (5.0%)  Neutral 0/20 (0%)  Disagree 19/20 (95.0%) | .610 | Agree 11/168 (6.5%)  Neutral 19/168 (11.3%)  Disagree 138/168 (82.1%) | .828 | Agree 2/25 (8.0%)  Neutral 3/25 (80.0%)  Disagree 20/25 (12.0%) | .711 |
|  | Post | Agree 0/5 (0%)  Neutral 0/5 (0%)  Disagree 5/5 (100%) |  | Agree 4/75 (5.3%)  Neutral 7/75 (9.3%)  Disagree 64/75 (85.3%) |  | Agree 0/8 (0%)  Neutral 1/8 (12.5%)  Disagree 7/8 (87.5%) |  |
| **I am concerned that I will be viewed negatively if the patient/family for whom I am providing care escalates concerns about clinical deterioration** | Pre | Agree 2/20 (10.0%)  Neutral 2/20 (10.0%)  Disagree 16/20 (80.0%) | .449 | Agree 26/167 (15.6%)  Neutral 28/167 (16.8%)  Disagree 113/167 (67.7%) | .270 | Agree 0/24 (0%)  Neutral 4/24 (16.7%)  Disagree 20/24 (83.3%) | .114 |
|  | Post | Agree 1/4 (25.0%)  Neutral 1/4 (25.0%)  Disagree 2/4 (50.0%) |  | Agree 6/75 (8.0%)  Neutral 13/75 (17.3%)  Disagree 56/75 (74.7%) |  | Agree 1/8 (12.5%)  Neutral 0/8 (0%)  Disagree 7/8 (87.5%) |  |
| **I am concerned that I will be viewed negatively if I escalated patient/family concerns about clinical deterioration with my senior staff** | Pre | Agree 1/19 (5.3%)  Neutral 3/19 (15.8%)  Disagree 15/19 (78.9%) | .347 | Agree 16/169 (9.5%)  Neutral 23/169 (13.6%)  Disagree 130/169 (76.9%) | .552 | Agree 2/24 (8.3%)  Neutral 3/24 (12.5%)  Disagree 19/24 (79.2%) | .309 |
|  | Post | Agree 1/4 (25.0%)  Neutral 0/4 (0%)  Disagree 3/4 (75.0%) |  | Agree 4/75 (5.3%)  Neutral 11/75 (14.7%)  Disagree 60/75 (80.0%) |  | Agree 2/8 (25.0%)  Neutral 0/8 (0%)  Disagree 6/8 (75.0%) |  |
| **I am aware of our LHN consumer escalation system** | Pre |  |  |  |  |  |  |
|  | Post | Yes 3/5 (60.0%) |  | Yes 63/75 (84.0%) |  | Yes 6/8 (75.0%) |  |
| **Impact of introducing a consumer escalation system: Increase workloads** | Pre | Agree 11/20 (55.0%)  Neutral 4/20 (20.0%)  Disagree 5/20 (25.0%) | .206 | Agree 73/170 (42.9%)  Neutral 46/170 (27.1%)  Disagree 51/170 (30.0%) | <.001 | Agree 6/24 (25.0%)  Neutral 7/24 (29.2%)  Disagree 11/24 (45.8%) | .343 |
|  | Post | Agree 1/3 (33.3%)  Neutral 2/3 (66.7%)  Disagree 0/3 (0%) |  | Agree 11/63 (17.5%)  Neutral 15/63 (23.8%)  Disagree 37/63 (58.7%) |  | Agree 0/6 (0%)  Neutral 3/6 (50.0%)  Disagree 3/6 (50.0%) |  |
| **Impact of introducing a consumer escalation system: Increase patient safety** | Pre | Agree 13/20 (65.0%)  Neutral 6/20 (30.0%)  Disagree 1/20 (5.0%) | .923 | Agree 137/170 (80.6%)  Neutral 26/170 (15.3%)  Disagree 7/170 (4.1%) | .195 | Agree 23/24 (95.8%)  Neutral 1/24 (4.2%)  Disagree 0/24 (0%) | .003 |
|  | Post | Agree 2/3 (66.7%)  Neutral 1/3 (33.3%)  Disagree 0/3 (0%) |  | Agree 44/63 (69.8%)  Neutral 14/63 (22.2%)  Disagree 5/63 (7.9%) |  | Agree 3/6 (50.0%)  Neutral 3/6 (50.0%)  Disagree 0/6 (0%) |  |
| **Impact of introducing a consumer escalation system: Promote patient centred care** | Pre | Agree 18/20 (90.0%)  Neutral 1/20 (5.0%)  Disagree 1/20 (5.0%) | .848 | Agree 146/170 (85.9%)  Neutral 18/170 (10.6%)  Disagree 6/170 (3.5%) | .035 | Agree 24/24 (100%)  Neutral 0/24 (0%)  Disagree 0/24 (0%) | .003 |
|  | Post | Agree 3/3 (100%)  Neutral 0/3 (0%)  Disagree 0/3 (0%) |  | Agree 46/63 (73.0%)  Neutral 10/63 (15.9%)  Disagree 7/63 (11.1%) |  | Agree 4/6 (66.7%)  Neutral 2/6 (33.3%)  Disagree 0/6 (0%) |  |
| **Impact of introducing a consumer escalation system: Promote patient-staff rapport** | Pre | Agree 14/20 (70.0%)  Neutral 4/20 (20.0%)  Disagree 2/20 (10.0%) | .772 | Agree 129/169 (76.3%)  Neutral 31/169 (18.3%)  Disagree 9/169 (5.3%) | .005 | Agree 20/24 (83.3%)  Neutral 4/24 (16.7%)  Disagree 0/24 (0%) | .001 |
|  | Post | Agree 2/3 (66.7%)  Neutral 1/3 (33.3%)  Disagree 0/3 (0%) |  | Agree 35/63 (55.6%)  Neutral 19/63 (30.2%)  Disagree 9/63 (14.3%) |  | Agree 1/6 (16.7%)  Neutral 5/6 (83.3%)  Disagree 0/6 (0%) |  |
| **Impact of introducing a consumer escalation system: Increase risk of generating patient-staff conflict** | Pre | Agree 10/20 (50.0%)  Neutral 4/20 (20.0%)  Disagree 6/20 (30.0%) | .158 | Agree 57/169 (33.7%)  Neutral 44/169 (26.0%)  Disagree 68/169 (40.2%) | .003 | Agree 4/24 (16.7%)  Neutral 8/24 (33.3%)  Disagree 12/24 (50.0%) | .535 |
|  | Post | Agree 0/3 (0%)  Neutral 2/3 (66.7%)  Disagree 1/3 (33.3%) |  | Agree 7/62 (11.3%)  Neutral 19/62 (30.6%)  Disagree 36/62 (58.1%) |  | Agree 0/6 (0%)  Neutral 2/6 (33.3%)  Disagree 4/6 (67.3%) |  |
| **Impact of introducing a consumer escalation system: No noticeable impact** | Pre |  |  |  |  |  |  |
|  | Post | Agree 0/3 (0%)  Neutral 2/3 (66.7%)  Disagree 1/3 (33.3%) |  | Agree 19/61 (31.1%)  Neutral 19/61 (31.1%)  Disagree 23/61 (37.7%) |  | Agree 2/6 (33.3%)  Neutral 4/6 (66.7%)  Disagree 0/6 (0%) |  |
| **Have you been involved in a situation where a patient/family expressed to you their concern with regard to acute clinical deterioration?** | Pre |  |  |  |  |  |  |
|  | Post | Yes 3/5 (60.0%) |  | Yes 42/76 (55.3%) |  | Yes 3/8 (37.5%) |  |
| **Action in response to concern: Reassured the patient/family** | Pre |  |  |  |  |  |  |
|  | Post | Yes 2/3 (66.7%) |  | Yes 38/42 (90.5%) |  | Yes 3/3 (100%) |  |
| **Action in response to concern: Increased frequency of observations** | Pre |  |  |  |  |  |  |
|  | Post | Yes 2/3 (66.7%) |  | Yes 38/42 (90.5%) |  | Yes 2/3 (66.7%) |  |
| **Action in response to concern: Notified a more senior nurse** | Pre |  |  |  |  |  |  |
|  | Post | Yes 1/3 (33.3%) |  | Yes 27/42 (64.3%) |  | Yes 2/3 (66.7%) |  |
| **Action in response to concern: Notified the admitting medical team** | Pre |  |  |  |  |  |  |
|  | Post | Yes 1/3 (33.3%) |  | Yes 36/42 (85.7%) |  | Yes 3/3 (100%) |  |
| **Action in response to concern: Notified the admitting medical Consultant** | Pre |  |  |  |  |  |  |
|  | Post | Yes 1/3 (33.3%) |  | Yes 12/42 (28.6%) |  | Yes 1/3 (33.3%) |  |
| **Action in response to concern: Triggered a RRT call** | Pre |  |  |  |  |  |  |
|  | Post | Yes 0/3 (0%) |  | Yes 9/42 (21.4%) |  | Yes 0/3 (0%) |  |

| **Pre and post agreement levels: Participant area of work** | | | | | | | | | |
| --- | --- | --- | --- | --- | --- | --- | --- | --- | --- |
| **Question/statement** |  | **Medical** | **p value** | **Surgical** | **p value** | **Critical Care** | **p value** | **Mental Health** | **p value** |
| **Patients and family members have a good knowledge of a patient's 'normal' clinical condition and behaviour** | Pre | Agree 107/118 (90.7%)  Neutral 10/118 (8.5%)  Disagree 1/118 (0.8%) | .847 | Agree 49/56 (87.5%)  Neutral 6/56 (10.7%)  Disagree 1/56 (1.8%) | .221 | Agree 35/40 (87.5%)  Neutral 5/40 (12.5%)  Disagree 0/40 (0%) | .024 |  |  |
|  | Post | Agree 27/29 (93.1%)  Neutral 2/29 (6.9%)  Disagree 0/29 (0%) |  | Agree 22/22 (100%)  Neutral 0/22 (0%)  Disagree 0/22 (0%) |  | Agree 5/6 (83.3%)  Neutral 0/6 (0%)  Disagree 1/6 (16.7%) |  | Agree 27/30 (90.0%)  Neutral 3/30 (10.0%)  Disagree 0/30 (0%) |  |
| **Patients and family members can be relied upon to detect changes in a patient's clinical condition indicative of acute deterioration** | Pre | Agree 64/118 (54.2%)  Neutral 39/118 (33.1%)  Disagree 15/118 (12.7%) | .002 | Agree 36/55 (65.5%)  Neutral 12/55 (21.8%)  Disagree 7/55 (12.7%) | .527 | Agree 20/40 (50.0%)  Neutral 13/40 (32.5%)  Disagree 7/40 (17.5%) | .280 |  |  |
|  | Post | Agree 26/29 (89.7%)  Neutral 3/29 (10.3%)  Disagree 0/29 (0%) |  | Agree 17/23 (73.9%)  Neutral 5/23 (21.7%)  Disagree 1/23 (4.3%) |  | Agree 5/6 (83.3%)  Neutral 1/6 (16.7%)  Disagree 0/6 (0%) |  | Agree 25/30 (83.3%)  Neutral 4/30 (13.3%)  Disagree 1/30 (3.3%) |  |
| **Patients and family members cannot be relied upon to recognise clinical deterioration in a patient's condition** | Pre | Agree 28/117 (23.9%)  Neutral 23/117 (19.7%)  Disagree 66/117 (56.4%) | .031 | Agree 10/55 (18.2%)  Neutral 9/55 (16.4%)  Disagree 36/55 (65.5%) | .762 | Agree 12/40 (30.0%)  Neutral 10/40 (25.0%)  Disagree 18/40 (45.0%) | .042 |  |  |
|  | Post | Agree 1/29 (3.4%)  Neutral 5/29 (17.2%)  Disagree 23/29 (79.3%) |  | Agree 3/23 (13.0%)  Neutral 3/23 (13.0%)  Disagree 17/23 (73.9%) |  | Agree 0/6 (0%)  Neutral 0/6 (0%)  Disagree 6/6 (100%) |  | Agree 8/30 (26.7%)  Neutral 6/30 (20.0%)  Disagree 16/30 (53.3%) |  |
| **Patients and family members are sufficiently confident to raise concerns about clinical deterioration in a patient's condition with the ward staff** | Pre | Agree 60/116 (51.7%)  Neutral 40/116 (34.5%)  Disagree 16/116 (13.8%) | .360 | Agree 28/56 (50.0%)  Neutral 22/56 (39.3%)  Disagree 6/56 (10.7%) | .092 | Agree 23/40 (57.5%)  Neutral 12/40 (30.0%)  Disagree 5/40 (12.5%) | .562 |  |  |
|  | Post | Agree 18/29 (62.1%)  Neutral 6/29 (20.7%)  Disagree 5/29 (17.2%) |  | Agree 8/23 (34.8%)  Neutral 8/23 (34.8%)  Disagree 7/23 (30.4%) |  | Agree 4/5 (80.0%)  Neutral 1/5 (20.0%)  Disagree 0/5 (0%) |  | Agree 17/30 (56.7%)  Neutral 5/30 (16.7%)  Disagree 8/30 (26.7%) |  |
| **If patient/family express concerns about deterioration in a patient’s condition, healthcare staff have a responsibility to listen** | Pre | Agree 117/117 (100%)  Neutral 0/117 (0%)  Disagree 0/117 (0%) | NA | Agree 56/56 (100%)  Neutral 0/56 (0%)  Disagree 0/56 (0%) | NA | Agree 40/40 (100%)  Neutral 0/40 (0%)  Disagree 0/40 (0%) | NA |  |  |
|  | Post | Agree 28/28 (100%)  Neutral 0/28 (0%)  Disagree 0/28 (0%) |  | Agree 23/23 (100%)  Neutral 0/23 (0%)  Disagree 0/23 (0%) |  | Agree 6/6 (100%)  Neutral 0/6 (0%)  Disagree 0/6 (0%) |  | Agree 30/30 (100%)  Neutral 0/30 (0%)  Disagree 0/30 (0%) |  |
| **If a patient or family member expresses concerns that a patient’s condition is deteriorating, healthcare staff should: Assess patient, including recording vital signs** | Pre | Agree 116/117 (99.1%)  Neutral 1/117 (0.9%)  Disagree 0/119 (0%) | .617 | Agree 56/56 (100%)  Neutral 0/56 (0%)  Disagree 0.56 (0%) | .116 | Agree 40/40 (100%)  Neutral 0/40 (0%)  Disagree 0/40 (0%) | .009 |  |  |
|  | Post | Agree 29/29 (100%)  Neutral 0/29 (0%)  Disagree 0/29 (0%) |  | Agree 22/23 (95.7%)  Neutral 0/23 (0%)  Disagree 1/23 (4.3%) |  | Agree 5/6 (83.3%)  Neutral 0/6 (0%)  Disagree 1/6 (16.7%) |  | Agree 29/29 (100%)  Neutral 0/29 (0%)  Disagree 0/29 (0%) |  |
| **If a patient or family member expresses concerns that a patient’s condition is deteriorating, healthcare staff should: Notify a senior nurse** | Pre |  |  |  |  |  |  |  |  |
|  | Post | Agree 24/29 (82.8%)  Neutral 3/29 (10.3%)  Disagree 2/29 (6.9%) |  | Agree 20/21 (95.2%)  Neutral 0/21 (0%)  Disagree 1/21 (4.8%) |  | Agree 5/6 (83.3%)  Neutral 0/6 (0%)  Disagree 1/6 (16.7%) |  | Agree 26/30 (86.7%)  Neutral 3/30 (10%)  Disagree 1/30 (3.3%) |  |
| **If a patient or family member expresses concerns that a patient’s condition is deteriorating, healthcare staff should: Notify the admitting medical team** | Pre | Agree 84/116 (72.4%)  Neutral 28/116 (24.1%)  Disagree 4/116 (3.4%) | 1.0 | Agree 44/55 (80.0%)  Neutral 9/55 (16.4%)  Disagree 2/55 (3.6%) | .219 | Agree 24/40 (60.0%)  Neutral 10/40 (25.0%)  Disagree 6/40 (15.0%) | .375 |  |  |
|  | Post | Agree 21/29 (72.4%)  Neutral 7/29 (24.1%)  Disagree 1/29 (3.4%) |  | Agree 14/21 (66.7%)  Neutral 4/21 (19.0%)  Disagree 3/21 (14.3%) |  | Agree 5/6 (83.3%)  Neutral 0/6 (0%)  Disagree 1/6 (16.7%) |  | Agree 26/29 (89.7%)  Neutral 2/29 (6.9%)  Disagree 1/29 (3.4%) |  |
| **If a patient or family member expresses concerns that a patient’s condition is deteriorating, healthcare staff should: Complete a medical or nursing review within 30 minutes** | Pre | Agree 101/113 (89.4%)  Neutral 7/113 (6.2%)  Disagree 5/113 (4.4%) | .820 | Agree 48/54 (88.9%)  Neutral 4/54 (7.4%)  Disagree 2/54 (3.7%) | .567 | Agree 28/40 (70.0%)  Neutral 8/40 (20.0%)  Disagree 4/40 (10.0%) | .462 |  |  |
|  | Post | Agree 27/29 (93.1%)  Neutral 1/29 (3.4%)  Disagree 1/29 (3.4%) |  | Agree 18/21 (85.7%)  Neutral 1/21 (4.8%)  Disagree 2/21 (9.5%) |  | Agree 5/6 (83.3%)  Neutral 0/6 (0%)  Disagree 1/6 (16.7%) |  | Agree 26/29 (89.7%)  Neutral 1/29 (3.4%)  Disagree 2/29 (6.9%) |  |
| **If patient/family concerns about clinical deterioration persist, the patient should be reviewed by a more senior clinician even if there is no evidence of deterioration as per the RDR chart** | Pre | Agree 95/116 (81.9%)  Neutral 19/116 (16.4%)  Disagree 2/116 (1.7%) | .142 | Agree 45/55 (81.8%)  Neutral 7/55 (12.7%)  Disagree 3/55 (5.5% | .864 | Agree 27/40 (67.5%)  Neutral 8/40 (20.0%)  Disagree 5/40 (12.5%) | .616 |  |  |
|  | Post | Agree 28/29 (96.6%)  Neutral 1/29 (3.4%)  Disagree 0/29 (0%) |  | Agree 18/23 (78.3%)  Neutral 3/23 (13.0%)  Disagree 2/23 (8.7%) |  | Agree 5/6 (83.3%)  Neutral 1/6 (16.7%)  Disagree 0/6 (0%) |  | Agree 27/29 (93.1%)  Neutral 2/29 (6.9%)  Disagree 0/29 (0%) |  |
| **If a patient or family member expresses concerns that a patient’s condition is deteriorating, healthcare staff should: Notify the admitting Medical Consultant/most senior medical officer** | Pre |  |  |  |  |  |  |  |  |
|  | Post | Agree 11/28 (39.3%)  Neutral 10/28 (35.7%)  Disagree 7/28 (25.0%) |  | Agree 4/19 (21.1%)  Neutral 8/19 (42.1%)  Disagree 7/19 (36.8%) |  | Agree 2/6 (33.3%)  Neutral 3/6 (50.0%)  Disagree 1/6 (16.7%) |  | Agree 17/28 (60.7%)  Neutral 10/28 (35.7%)  Disagree 1/28 (3.6%) |  |
| **If patient/family concerns about clinical deterioration persist despite senior nurse and RMO/Registrar review, escalation should occur to the treating Consultant/most senior medical officer, even if there is no evidence of deterioration as per the RDR chart** | Pre | Agree 62/118 (52.5%)  Neutral 45/118 (38.1%)  Disagree 11/118 (9.3%) | .734 | Agree 35/56 (62.5%)  Neutral 17/56 (30.4%)  Disagree 4/56 (7.1%) | .254 | Agree 16/40 (40.0%)  Neutral 13/40 (32.5%)  Disagree 11/40 (27.5%) | .286 |  |  |
|  | Post | Agree 17/28 (60.7%)  Neutral 9/28 (32.1%)  Disagree 2/28 (7.1%) |  | Agree 15/23 (65.2%)  Neutral 4/23 (17.4%)  Disagree 4/23 (17.4%) |  | Agree 4/6 (66.7%)  Neutral 2/6 (33.3%)  Disagree 0/6 (0%) |  | Agree 23/30 (76.7%)  Neutral 4/30 (13.3%)  Disagree 3/30 (10.0%) |  |
| **If a patient or family member expresses concerns that a patient’s condition is deteriorating, healthcare staff should: Trigger a RRT activation** | Pre |  |  |  |  |  |  |  |  |
|  | Post | Agree 11/27 (40.7%)  Neutral 8/27 (29.6%)  Disagree 8/27 (29.6%) |  | Agree 9/21 (42.9%)  Neutral 5/21 (23.8%)  Disagree 7/21 (33.3%) |  | Agree 3/6 (50.0%)  Neutral 2/6 (33.3%)  Disagree 1/6 (16.7%) |  | Agree 15/26 (57.7%)  Neutral 10/26 (38.5%)  Disagree 1/26 (3.8%) |  |
| **If a patient or family member asked me to call the RRT, I would not hesitate to do so** | Pre |  |  |  |  |  |  |  |  |
|  | Post | Agree 10/28 (35.7%)  Neutral 9/28 (32.1%)  Disagree 9/28 (32.1%) |  | Agree 7/23 (30.4%)  Neutral 7/23 (30.4%)  Disagree 9/23 (39.1%) |  | Agree 3/6 (50.0%)  Neutral 1/6 (16.7%)  Disagree 2/6 (33.3%) |  | Agree 13/30 (43.3%)  Neutral 11/30 (36.7%)  Disagree 6/30 (20.0%) |  |
| **Patients and family members should be encouraged to escalate concerns to the ward staff about clinical deterioration in a patient's condition** | Pre | Agree 116/117 (99.1%)  Neutral 1/117 (0.9%)  Disagree 0/117 (0%) | .282 | Agree 56/56 (100%)  Neutral 0/56 (0%)  Disagree 0/56 (0%) | .082 | Agree 36/38 (94.7%)  Neutral 1/38 (2.6%)  Disagree 1/38 (2.6%) | .848 |  |  |
|  | Post | Agree 28/29 (96.6%)  Neutral 1/29 (3.4%)  Disagree 0/29 (0%) |  | Agree 21/23 (91.3%)  Neutral 1/23 (4.3%)  Disagree 1/23 (4.3%) |  | Agree 6/6 (100%)  Neutral 0/6 (0%)  Disagree 0/6 (0%) |  | Agree 29/30 (96.7%)  Neutral 0/30 (0%)  Disagree 1/30 (3.3%) |  |
| **Patients and family members should be able to bypass the ward staff and directly trigger a RRT if concerned about clinical deterioration in a patient's condition** | Pre | Agree 18/117 (15.4%)  Neutral 21/117 (17.9%)  Disagree 78/117 (66.7%) |  | Agree 4/55 (7.3%)  Neutral 20/55 (36.4%)  Disagree 31/55 (56.4%) |  | Agree 4/39 (10.3%)  Neutral 3/39 (7.7%)  Disagree 32/39 (82.1%) |  |  |  |
|  | Post |  |  |  |  |  |  |  |  |
| **Staff concern, or ‘worried’ is a valid RRT calling criteria** | Pre | Agree 99/117 (84.6%)  Neutral 7/117 (6.0%)  Disagree 11/117 (9.4%) | .346 | Agree 45/56 (80.4%)  Neutral 8/56 (14.3%)  Disagree 3/56 (5.4%) | .788 | Agree 33/40 (82.5%)  Neutral 5/40 (12.5%)  Disagree 2/40 (5.0%) | .538 |  |  |
|  | Post | Agree 23/29 (79.3%)  Neutral 4/29 (13.8%)  Disagree 2/29 (6.9%) |  | Agree 17/23 (73.9%)  Neutral 4/23 (17.4%)  Disagree 2/23 (8.7%) |  | Agree 6/6 (100%)  Neutral 0/6 (0%)  Disagree 0/6 (0%) |  | Agree 23/30 (76.7%)  Neutral 5/30 (16.7%)  Disagree 2/30 (6.7%) |  |
| **You have no hesitations to trigger a RRT call if you are ‘worried’** | Pre | Agree 86/116 (74.1%)  Neutral 15/116 (12.9%)  Disagree 15/116 (12.9%) | .665 | Agree 41/56 (73.2%)  Neutral 12/56 (21.4%)  Disagree 3/56 (5.4%) | .489 | Agree 33/39 (84.6%)  Neutral 1/39 (2.6%)  Disagree 5/39 (12.8%) | .587 |  |  |
|  | Post | Agree 23/29 (79.3%)  Neutral 4/29 (13.8%)  Disagree 2/29 (6.9%) |  | Agree 16/23 (69.6%)  Neutral 4/23 (17.4%)  Disagree 3/23 (13.0%) |  | Agree 6/6 (100%)  Neutral 0/6 (0%)  Disagree 0/6 (0%) |  | Agree 22/30 (73.3%)  Neutral 5/30 (16.7%)  Disagree 3/30 (10.0%) |  |
| **If there is no evidence of deterioration as per the RDR chart, then there is no reason to escalate clinical concern** | Pre | Agree 6/116 (5.2%)  Neutral 15/116 (12.9%)  Disagree 95/116 (81.9%) | .763 | Agree 2/56 (3.6%)  Neutral 9/56 (16.1%)  Disagree 45/56 (80.4%) | .287 | Agree 5/38 (13.2%)  Neutral 2/38 (5.3%)  Disagree 31/38 (81.6%) | .411 |  |  |
|  | Post | Agree 2/29 (6.9%)  Neutral 5/29 (17.2%)  Disagree 22/29 (75.9%) |  | Agree 3/23 (13.0%)  Neutral 3/23 (13.0%)  Disagree 17/23 (73.9%) |  | Agree 0/6 (0%)  Neutral 1/6 (16.7%)  Disagree 5/6 (83.3%) |  | Agree 2/30 (6.7%)  Neutral 3/30 (10.0%)  Disagree 25/30 (83.3%) |  |
| **If there is no evidence of deterioration as per the RDR chart, then there is no reason for patients/family to be concerned** | Pre | Agree 6/116 (5.2%)  Neutral 9/116 (7.8%)  Disagree 101/116 (87.1%) | .426 | Agree 1/56 (1.8%)  Neutral 9/56 (16.1%)  Disagree 46/56 (82.1%) | .790 | Agree 6/40 (15.0%)  Neutral 4/40 (10.0%)  Disagree 30/40 (75.0%) | .384 |  |  |
|  | Post | Agree 0/29 (0%)  Neutral 3/29 (10.3%)  Disagree 26/29 (89.7%) |  | Agree 1/23 (4.3%)  Neutral 4/23 (17.4%)  Disagree 18/23 (78.3%) |  | Agree 0/6 (0%)  Neutral 0/6 (0%)  Disagree 6/6 (100%) |  | Agree 3/29 (10.3%)  Neutral 1/29 (3.4%)  Disagree 25/29 (86.2%) |  |
| **I am concerned that I will be viewed negatively if the patient/family for whom I am providing care escalates concerns about clinical deterioration** | Pre | Agree 15/117 (12.8%)  Neutral 17/117 (14.5%)  Disagree 85/117 (72.6%) | .615 | Agree 4/55 (7.3%)  Neutral 10/55 (18.2%)  Disagree 41/55 (74.5%) | .278 | Agree 8/38 (21.1%)  Neutral 7/38 (18.4%)  Disagree 23/38 (60.5%) | .395 |  |  |
|  | Post | Agree 3/27 (11.1%)  Neutral 6/27 (22.2%)  Disagree 18/27 (66.7%) |  | Agree 2/23 (8.7%)  Neutral 1/21 (4.3%)  Disagree 20/23 (87.0%) |  | Agree 0/6 (0%)  Neutral 2/6 (33.3%)  Disagree 4/6 (66.7%) |  | Agree 3/30 (10.0%)  Neutral 5/30 (16.7%)  Disagree 22/30 (73.3%) |  |
| **I am concerned that I will be viewed negatively if I escalated patient/family concerns about clinical deterioration with my senior staff** | Pre | Agree 10/116 (8.6%)  Neutral 11/116 (9.5%)  Disagree 95/116 (81.9%) | .146 | Agree 4/56 (7.1%)  Neutral 12/56 (21.4%)  Disagree 40/56 (71.4%) | .177 | Agree 4/39 (10.3%)  Neutral 6/39 (15.4%)  Disagree 29/39 (74.4%) | .713 |  |  |
|  | Post | Agree 1/27 (3.7%)  Neutral 6/27 (22.2%)  Disagree 20/27 (74.1%) |  | Agree 2/23 (8.7%)  Neutral 1/23 (4.3%)  Disagree 20/23 (87.0%) |  | Agree 0/6 (0%)  Neutral 1/6 (16.7%)  Disagree 5/6 (83.3%) |  | Agree 3/30 (10.0%)  Neutral 3/30 (10.0%)  Disagree 24/30 (80.0%) |  |
| **I am aware of our LHN consumer escalation system** | Pre |  |  |  |  |  |  |  |  |
|  | Post | Yes 23/29 (79.3%) |  | Yes 18/22 (81.8%) |  | Yes 6/6 (100%) |  | Yes 24/30 (80.0%) |  |
| **Impact of introducing a consumer escalation system: Increase workloads** | Pre | Agree 48/117 (41.0%)  Neutral 38/117 (35.2%)  Disagree 31/117 (26.5%) | .054 | Agree 19/56 (33.9%)  Neutral 12/56 (21.4%)  Disagree 25/56 (44.6%) | .148 | Agree 22/40 (55.0%)  Neutral 7/40 (17.5%)  Disagree 11/40 (27.5%) | .512 |  |  |
|  | Post | Agree 4/23 (17.4%)  Neutral 8/23 (34.8%)  Disagree 11/23 (47.8%) |  | Agree 2/18 (11.1%)  Neutral 4/18 (22.2%)  Disagree 12/18 (66.7%) |  | Agree 2/6 (33.3%)  Neutral 1/6 (16.7%)  Disagree 3/6 (50.0%) |  | Agree 4/24 (16.7%)  Neutral 6/24 (25.0%)  Disagree 14/24 (58.3%) |  |
| **Impact of introducing a consumer escalation system: Increase patient safety** | Pre | Agree 92/117 (78.6%)  Neutral 20/117 (17.1%)  Disagree 5/117 (4.3%) | .552 | Agree 51/56 (91.1%)  Neutral 5/56 (8.9%)  Disagree 0/56 (0%) | .126 | Agree 29/40 (72.5%)  Neutral 8/40 (20.0%)  Disagree 3/40 (7.5%) | .247 |  |  |
|  | Post | Agree 16/23 (69.6%)  Neutral 5/23 (21.7%)  Disagree 2/23 (8.7%) |  | Agree 14/18 (77.8%)  Neutral 3/18 (16.7%)  Disagree 1/18 (5.60%) |  | Agree 3/6 (50.0%)  Neutral 3/6 (50.0%)  Disagree 0/6 (0%) |  | Agree 15/24 (62.5%)  Neutral 7/24 (29.2%)  Disagree 2/24 (8.3%) |  |
| **Impact of introducing a consumer escalation system: Promote patient centred care** | Pre | Agree 105/117 (89.7%)  Neutral 9/117 (7.7%)  Disagree 3/117 (2.6%) |  | Agree 50/56 (89.3%)  Neutral 5/56 (8.9%)  Disagree 1/56 (1.8%) |  | Agree 32/40 (80.0%)  Neutral 5/40 (12.5%)  Disagree 3/40 (7.5%) |  |  |  |
|  | Post | Agree 16/23 (69.6%)  Neutral 5/23 (21.7%)  Disagree 2/23 (8.7%) | .035 | Agree 13/18 (72.2%)  Neutral 2/18 (11.1%)  Disagree 3/18 (16.7%) | .047 | Agree 4/6 (66.7%)  Neutral 1/6 (16.7%)  Disagree 1/6 (16.7%) | .707 | Agree 19/24 (79.2%)  Neutral 4/24 (16.7%)  Disagree 1/24 (4.2%) |  |
| **Impact of introducing a consumer escalation system: Promote patient-staff rapport** | Pre | Agree 91/116 (78.4%)  Neutral 21/116 (18.1%)  Disagree 4/116 (3.4%) | .045 | Agree 46/56 (82.1%)  Neutral 9/56 (16.1%)  Disagree 1/56 (1.8%) | .007 | Agree 25/40 (62.5%)  Neutral 9/40 (22.5%)  Disagree 6/40 (15.0%) | .819 |  |  |
|  | Post | Agree 13/23 (56.5%)  Neutral 7/23 (30.4%)  Disagree 3/23 (13.0%) |  | Agree 10/18 (55.6%)  Neutral 4/18 (22.2%)  Disagree 4/18 (22.2%) |  | Agree 3/6 (50.0%)  Neutral 2/6 (33.3%)  Disagree 1/6 (16.7%) |  | Agree 11/24 (45.8%)  Neutral 12/24 (50.0%)  Disagree 1/24 (4.2%) |  |
| **Impact of introducing a consumer escalation system: Increase risk of generating patient-staff conflict** | Pre | Agree 45/116 (38.8%)  Neutral 24/116 (20.7%)  Disagree 47/116 (40.5%) | .005 | Agree 11/56 (19.6%)  Neutral 19/56 (33.9%)  Disagree 26/56 (46.4%) | .284 | Agree 14/40 (35.0%)  Neutral 13/40 (32.5%)  Disagree 13/40 (32.5%) | .640 |  |  |
|  | Post | Agree 2/23 (8.7%)  Neutral 11/23 (47.8%)  Disagree 10/23 (43.5%) |  | Agree 3/18 (16.7%)  Neutral 3/18 (16.7%)  Disagree 12/18 (66.7%) |  | Agree 2/6 (33.3%)  Neutral 1/6 (16.7%)  Disagree 3/6 (50.0%) |  | Agree 0/23 (0%)  Neutral 7/23 (30.4%)  Disagree 16/23 (69.6%) |  |
| **Impact of introducing a consumer escalation system: No noticeable impact** | Pre |  |  |  |  |  |  |  |  |
|  | Post | Agree 7/22 (31.8%)  Neutral 9/22 (40.9%)  Disagree 6/22 (27.3%) |  | Agree 5/18 (27.8%)  Neutral 6/18 (33.3%)  Disagree 7/18 (38.9%) |  | Agree 2/6 (33.3%)  Neutral 1/6 (16.7%)  Disagree 3/6 (50.0%) |  | Agree 7/23 (30.4%)  Neutral 9/23 (39.1%)  Disagree 7/23 (30.4%) |  |
| **Have you been involved in a situation where a patient/family expressed to you their concern with regard to acute clinical deterioration?** | Pre |  |  |  |  |  |  |  |  |
|  | Post | Yes 15/29 (51.7%) |  | Yes 12/23 (52.2%) |  | Yes 4/6 (66.7%) |  | Yes 16/30 (53.3%) |  |
| **Action in response to concern: Reassured the patient/family** | Pre |  |  |  |  |  |  |  |  |
|  | Post | Yes 14/15 (93.3%) |  | Yes 10/12 (83.3%) |  | Yes 3/4 (75.0%) |  | Yes 15/16 (93.8%) |  |
| **Action in response to concern: Increased frequency of observations** | Pre |  |  |  |  |  |  |  |  |
|  | Post | Yes 14/15 (93.3%) |  | Yes 10/12 (83.3%) |  | Yes 3/4 (75.0%) |  | Yes 14/16 (87.5%) |  |
| **Action in response to concern: Notified a more senior nurse** | Pre |  |  |  |  |  |  |  |  |
|  | Post | Yes 11/15 (73.3%) |  | Yes 3/12 (25.0%) |  | Yes 3/4 (75.0%) |  | Yes 12/16 (75.0%) |  |
| **Action in response to concern: Notified the admitting medical team** | Pre |  |  |  |  |  |  |  |  |
|  | Post | Yes 10/15 (66.7%) |  | Yes 11/12 (91.7%) |  | Yes 4/4 (100%) |  | Yes 14/16 (87.5%) |  |
| **Action in response to concern: Notified the admitting medical Consultant** | Pre |  |  |  |  |  |  |  |  |
|  | Post | Yes 4/15 (26.7%) |  | Yes 2/12 (16.7%) |  | Yes 2/4 (50.0%) |  | Yes 6/16 (37.5%) |  |
| **Action in response to concern: Triggered a RRT call** | Pre |  |  |  |  |  |  |  |  |
|  | Post | Yes 3/15 (20.0%) |  | Yes 2/12 (16.7%) |  | Yes 1/4 (25.0%) |  | Yes 3/16 (18.8%) |  |
